# Complementing aculiferan mitogenomics: comparative characterization of mitochondrial genomes of Solenogastres (Mollusca, Aplacophora)

**DOI:** 10.1186/s12862-024-02311-5

**Published:** 2024-10-18

**Authors:** Franziska S. Bergmeier, Andreas Brachmann, Kevin M. Kocot, Francesca Leasi, Albert J. Poustka, Michael Schrödl, Joseph L. Sevigny, W. Kelley Thomas, Christiane Todt, Katharina M. Jörger

**Affiliations:** 1 Faculty of Biology, Ludwig-Maximilians-Universität München, Systematic Zoology, Munich, Germany; 2 Faculty of Biology, Genetics, Ludwig-Maximilians-Universität München, Munich, Germany; 3 Department of Biological Sciences and Alabama Museum of Natural History, University of Alabama, Tuscaloosa, AL USA; 4 Department of Biology, Geology, and Environmental Science, University of Tennessee at Chattanooga, Chattanooga, TN USA; 5 Dahlem Centre for Genome Research and Medical Systems Biology, Environmental and Phylogenomics Group, Berlin, Germany; 6 Stiftung Naturschutz Berlin, Berlin, Germany; 7 SNSB-Bavarian State Collection of Zoology, Munich, Germany; 8 Hubbard Centre for Genome Studies, University of New Hampshire, Durham, NH USA; 9 Biota Naturkompetanse AS, Bergen, Norway

**Keywords:** Mollusca, Neomeniomorpha, Gene arrangement, Ancestral gene order, Mitochondrial genome, Aculifera, Aplacophora

## Abstract

**Background:**

With the advances in high-throughput sequencing and bioinformatic pipelines, mitochondrial genomes have become increasingly popular for phylogenetic analyses across different clades of invertebrates. Despite the vast rise in available mitogenomic datasets of molluscs, one class of aplacophoran molluscs – Solenogastres (or Neomeniomorpha) – is still neglected.

**Results:**

Here, we present six new mitochondrial genomes from five families of Solenogastres (Amphimeniidae, Gymnomeniidae, Proneomeniidae, Pruvotinidae, Simrothiellidae), including the first complete mitogenomes, thereby now representing three of the four traditional orders. Solenogaster mitogenomes are variable in size (ranging from approximately 15,000 bp to over 17,000 bp). The gene order of the 13 protein coding genes and two rRNA genes is conserved in three blocks, but considerable variation occurs in the order of the 22 tRNA genes. Based on phylogenetic analyses and reconstruction of ancestral mitochondrial genomes of Aculifera, the position of (1) trnD gene between atp8 and atp6, (2) trnT and P genes between atp6 and nad5, and (3) trnL1 gene between G and E, resulting in a ‘MCYWQGL1E’-block of tRNA genes, are all three considered synapomorphies for Solenogastres. The tRNA gene block ‘KARNI’ present in Polyplacophora and several conchiferan taxa is dissolved in Solenogastres.

**Conclusion:**

Our study shows that mitogenomes are suitable to resolve the phylogenetic relationships among Aculifera and within Solenogastres, thus presenting a cost and time efficient compromise to approach evolutionary history in these clades.

**Supplementary Information:**

The online version contains supplementary material available at 10.1186/s12862-024-02311-5.

## Background

Significant advancements in sequencing technology and data mining have resulted in a remarkable increase in the availability of mitochondrial genomes (mitogenomes) in recent decades, revealing a great diversity in genome size and genome architecture across various phyla of Metazoa. Bilaterian animals usually have highly compact (approximately 16 kb) and circular mitogenomes, comprising a standard set of 13 protein coding genes (PCGs), two ribosomal RNA (rRNA) genes and 22 transfer RNA (tRNA) genes [1]. However, there are a few exceptions such as the absence of atp8 in nematodes and flatworms [2, 3]. Several major groups of Metazoa have been recognized to have a high consistency in mitochondrial gene order with only few rearrangements [4], but even in intensively studied vertebrate mitogenomes this presumed conservation [5] has been questioned recently, revealing numerous (mainly tRNA related) rearrangements [6]. Molluscan mitogenomes in particular defy classic textbook concepts by displaying an exceptionally high degree of diversity: Molluscan mitogenomes vary tremendously in size, from compact genomes of heterobranch gastropods which are 13.6 kb in length to the largest known animal mitogenome found in a scallop, which exceeds 50 kb [7, 8]. Gene rearrangements, including those involving PCGs and rRNA genes have been reported across most major molluscan groups (see e.g [9]. and references therein), likely mediated through ‘tandem duplication random losses’ of tRNA genes [10]. Even instances of gene duplications have been documented, e.g., as much as six duplicated genes in species of deep-sea squid [11] and a putative loss of atp8 in some bivalves [12]. Lastly, regarding mitochondrial inheritance, atypical patterns featuring the transmission of mtDNAs from both maternal and paternal lineages occur in certain bivalves (termed doubly uniparental inheritance – DUI [9, 13]).

Increase in data availability and enhanced bioinformatic pipelines have also increased the use of mitogenomes in phylogenetic studies. Mitogenomic data have helped support novel evolutionary hypotheses and taxonomic revisions across various groups of invertebrates like arthropods, annelids, echinoderms and molluscs (e.g [14–17]. Due to the exceptionally high degree of rearrangements in molluscan mitogenome evolution and high substitution rates, the use of mitogenomes to resolve deep molluscan relationships has seen little promise, hampered by convergent evolution and long branch attraction in phylogenetic analyses (see e.g [18–20]). In contrast, at lower taxonomic level with denser taxon sampling, mitogenomes have delivered promising results to elucidate phylogenetic relationships [9, 21–24]. By now, a little over 1,250 molluscan mitochondrial genomes are currently available in NCBI’s Nucleotide database (accessed 21th of January 2024, search term: “Mollusca mitochondrion complete genome”), with the vast majority belonging to well-known classes like Cephalopoda, Bivalvia, and Gastropoda [9]. While mitogenomes of minor molluscan classes like Monoplacophora, Caudofoveata and Scaphopoda have been sequenced and comparatively studied [19, 22, 25–29], aplacophoran Solenogastres still lack a complete annotated mitochondrial genome. With only one incomplete (*Neomenia carinata* from Mikkelsen et al. [22]) and one unverified and unpublished mitochondrial genome (*Epimenia babai*, GenBank accession number MT798543.1) available on GenBank [22], the contribution of this little-known class of molluscs to the diversity of molluscan mitogenomes remains unknown, and its potential towards a better understanding of the complex evolution of mitochondrial genomes in molluscs remains unexplored.

This shortcoming is particularly critical as Solenogastres play a crucial role in our understanding of deep molluscan evolution: In contrast to earlier views on molluscan evolution based on morphological data, which placed Solenogastres and Caudofoveata (either as a monophylum or grade) at the base of the molluscan tree [30–32] modern phylogenomic analyses consistently converge towards the Conchifera-Aculifera hypothesis, i.e., a basal dichotomy between primarily shell-bearing (Conchifera) and spicule-bearing molluscs (Aculifera, with Polyplacophora as sister group to aplacophoran Caudofoveata and Solenogastres) [33–35]. This evolutionary scenario is further supported by comparative developmental and gene expression studies [36, 37]. Therefore, analyzing the diversity of solenogaster mitogenomes might impact our understanding of ancestral gene arrangements of Aculifera and provide another step towards a solid hypothesis on the evolution of molluscan mitogenomes.

Moreover, phylogenetic relationships of Solenogastres have been shown to be at odds with the established systematics of the group. Over the past 150 years, around 300 species of this class have been formally described and classified based mainly on external morphology (i.e., habitus and scleritome) and internal anatomy (among others characters of the reproductive system, different glands associated with the digestive system (for monographies and systematics see [38–41])). However, morphocladistic approaches have been unable to resolve internal relationships of Solenogastres, likely due to the high degree of convergent evolution found in these worm-shaped molluscs [42]. Recent phylogenetic studies based on two mitochondrial markers [43] and transcriptomic data [44, 45] have revealed conflicts in traditional systematics, such as rendering one of the main orders (Cavibelonia Salvini-Plawen, 1978) paraphyletic and presenting a new hypothesis on the sister group to all other remaining Solenogastres (family Amphimeniidae Salvini-Plawen, 1972) [43, 44] (but see [45] for alternative hypotheses). Each of those phylogenetic approaches has their strengths and weaknesses: Transcriptomic analyses are powerful in resolving phylogenetic relationships among Solenogastres [44], but this approach is potentially more costly and bioinformatically challenging, limiting their application across large-scale taxon sampling. On the other hand, multi marker barcoding approaches are easily accessible and cost-efficient allowing for dense taxon sampling, but often provide poor resolution for deeper nodes [43, 46]. In Solenogastres, sequencing of additional nuclear markers is hampered by secondary structures and generally results in the amplification of exogenous DNA, i.e. gut contents [47–49].

In this study, we aim to explore the potential of mitogenomics as an intermediate solution for resolving the phylogenetic relationships of Solenogastres (see [50] for mitogenomes as a reliable compromise in non-model taxa). We provide the first complete mitochondrial genomes of Solenogastres, generated from six species representing the two most speciose orders “Cavibelonia” Salvini-Plawen, 1978 and Pholidoskepia Salvini-Plawen, 1978. We comparatively analyze their genomic architecture and investigate taxon-specific modifications such as gene (re)arrangements. We explore whether mitogenomic gene arrangements provide (syn)apomorphic characters useful for phylogenetic inference and investigate if protein coding genes and ribosomal RNA genes can potentially result in well-resolved tree topologies.

By complementing data on this still neglected class of molluscs to the existing knowledge of molluscan mitogenome diversity, we aim to critically reassess previous hypotheses on ancestral mitogenome order in major molluscan clades, establish a hypothesis on the ancestral solenogaster mitogenome, and discuss its potential impact on the ancestral aculiferan mitogenome.

## Results

### Characteristics of solenogaster mitochondrial genomes

**Fig. 1:**
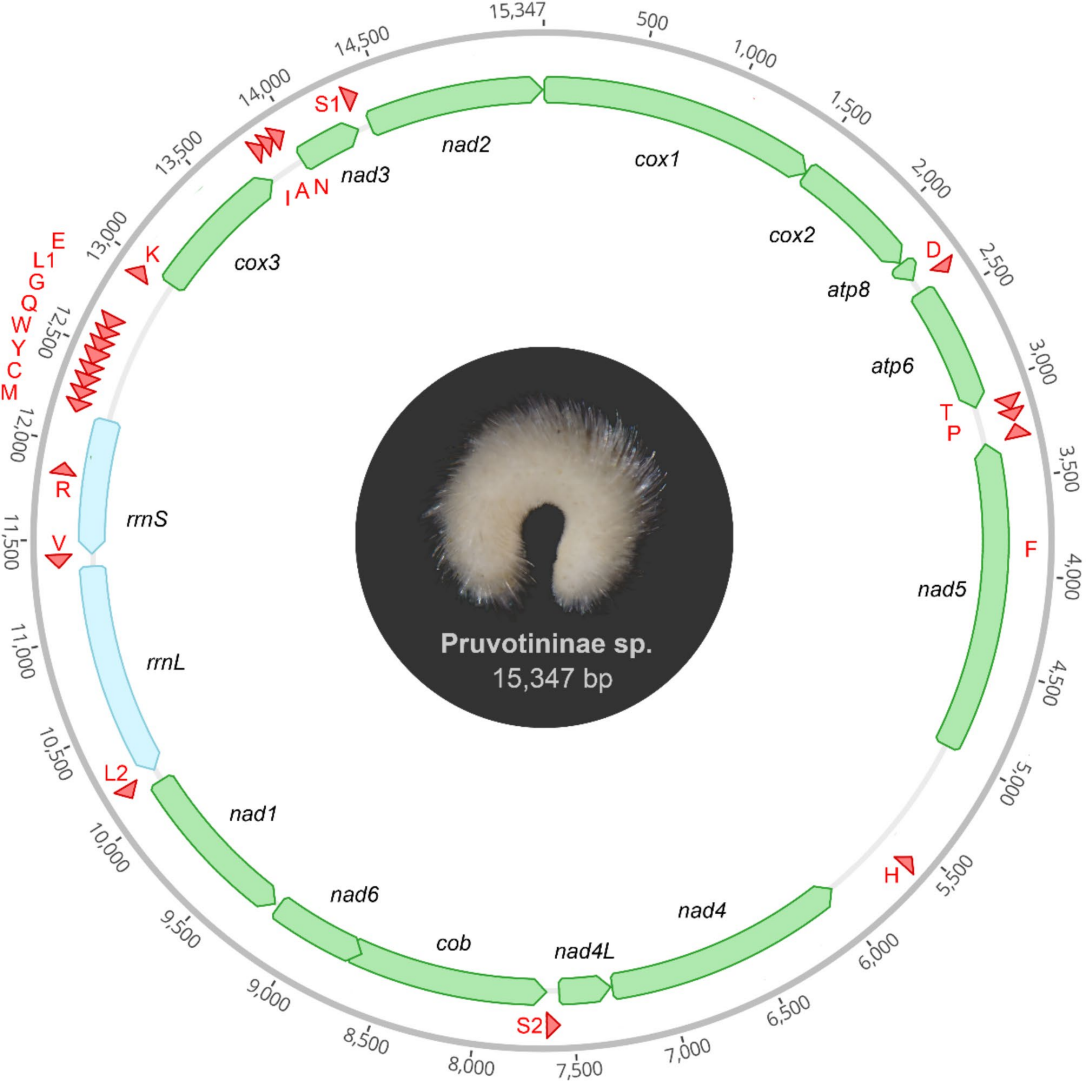
Illustration of the circularized mitochondrial genome of Pruvotininae sp. (Pruvotinidae). Arrows indicate direction of transcription. Protein coding genes in green, ribosomal RNA in blue, transfer RNA in red. Body size of animal approx. 2 mm

**Fig. 2:**
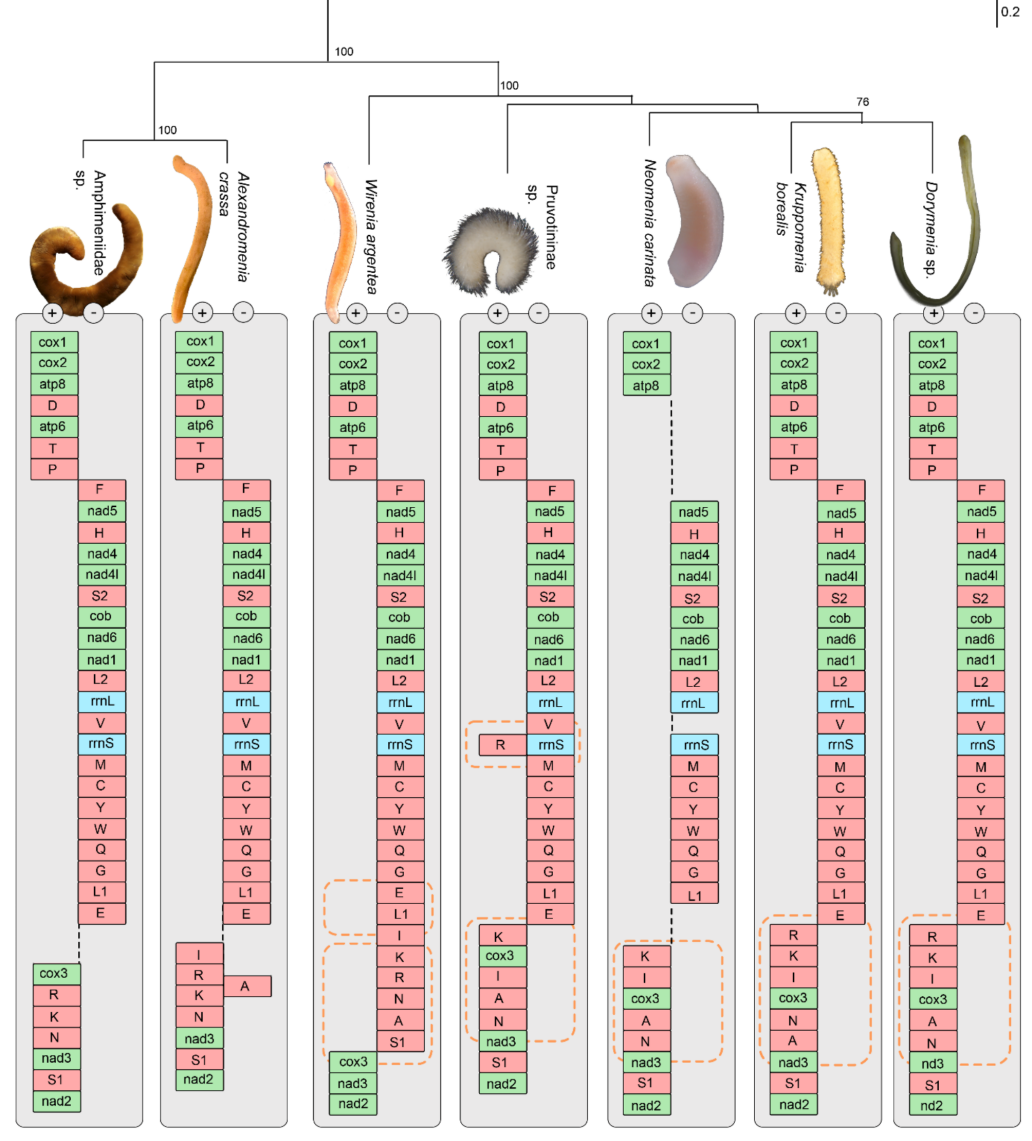
Maximum-likelihood tree of Solenogastres (based on 13 protein coding genes translated into amino acids) showing respective mitogenomic gene arrangements. Dotted orange boxes indicate blocks with variations in gene arrangement, dotted black lines indicate missing sections. Protein coding genes in green, ribosomal RNA in blue, transfer RNA in red. Body sizes: Amphimeniidae sp., 62 mm; *Alexandromenia crassa*, 21 mm, *Wirenia argentea*, 6 mm, Pruvotininae sp., 2 mm, *Neomenia carinata* 15 mm, *Kruppomenia borealis*, 8 mm, *Dorymenia* sp., 50 mm

The complete mitochondrial genome of Pruvotininae sp. is 15,347 base pairs (bp) long and contains the standard set of 13 protein coding genes (PCGs), two rRNA genes, and 22 tRNA genes (Fig. 1). Other analyzed mitogenomes range in size from 15,103 (*Dorymenia* sp.) to 17,090 bp (*Wirenia argentea*) (Table 1). All complete mitogenomes consist of the standard set of 37 genes (Fig. 2, see also Supplementary Table 1).

**Table 1:** Solenogaster mitogenomes used in the present study and associated sampling data

Taxon	Nr. of mt contigs and total length	Voucher number	Sampling data
*Alexandromenia crassa*	Two, 15,748 bp	n.a.	Kobbaleia, Bergen, Norway. UB field course, 2009.
Amphimeniidae sp.	Two, 15,587 bp	ZSM Mol 20190581	Northwest Pacific, KuramBio II cruise, St. 5, 2016. 7,154 m.
*Dorymenia* sp.	Two, 15,103 bp	ZSM Mol 20240448	Southern Ocean, Systco II cruise ANT XXVIII/3 RV Polarstern, St. PS79/141-08, 2012. 4,112 m.
Pruvotininae sp.	One, 15,347 bp	ZSM Mol 20090329	Antarctica. Andeep-Systco cruise ANT XXIV/2 RV Polarstern, St. PS71/048 − 01, 2007. 590 m.
*Kruppomenia borealis*	Two, 15,857 bp	ALMNH: Inv:25751	Haugolandsosen (near Bergen, Norway), UB collecting cruise, 2006. 180–220 m.
*Wirenia argentea*	Two, 17,090 bp	ALMNH: Inv:25752	Haugolandsosen (near Bergen, Norway), UB collecting cruise, 2012. 180–220 m.

**Table legend**: Abbreviations of natural history collections for voucher deposition: ALMNH-Inv, Invertebrate Collection of the Natural History Museum of Alabama (USA), UB, University of Bergen (Norway), ZSM Mol, Mollusca Collection of the SNSB-Bavarian State Collection of Zoology (Germany). n.a., not available. Sampling data includes (if available) locality, cruise, station, year, depth

The GC contents in complete mitogenomes range from 23.4% in *Wirenia argentea* to 31.3% in *Kruppomenia borealis*, indicating a nucleotide compositions bias towards A + T. Most complete mitochondrial genomes exhibit negative AT-skews (ranging from 0.0813 in *W. argentea* to -0.1772 in *Dorymenia* sp.) and positive GC-skews (from 0.1562 in *W. argentea* to 0.2812 in *Dorymenia* sp.), except for Pruvotininae sp. which shows slightly positive AT- and GC-skews (0.0057 / 0.00124) (see supplementary Table 2). T and G skew is present in all PCGs. PCGs encoded on the forward strand are also skewed towards T and G while PCGs of the reverse strand are skewed towards T and C, except for Pruvotininae sp. and *Dorymenia* sp. with a skew towards T + G, respectively A + C on the reverse strand (see supplementary Table 2). Across PCGs of all complete investigated mitogenomes, A + T contents of the third codon positions are highest (72.6 − 84.7%), followed by the second (64.2 − 69.8%) and first codon positions (61.5 − 76.3%) (supplementary Table 2).

The distribution of PCGs is nearly equal between the two strands and follows a consistent distribution across all analyzed Solenogastres. The cytochrome *c* oxidase subunits (cox1, cox2, cox3), ATP synthase subunits (atp6, atp8), and NADH dehydrogenase subunits (nad2 and nad3) are located on the plus strand, while the remaining nads (nad1, nad4, nad4L, nad5 and nad6) along with cytochrome *b* (cob) are located on the minus strand (Fig. 2). Both ribosomal genes (rrnS and rrnL) are located on the minus strand, and between nine to ten of the typical 22 tRNA genes are situated on the plus strand, with the rest on the minus strand. However, in *Wirenia argentea* tRNAs I, K, R, N, A, and S1 genes are reversed to the minus strand forming a cluster of 14 tRNAs in total, leaving only three tRNA genes (D, T, P) on the plus strand (Fig. 2).

**Fig. 3:**
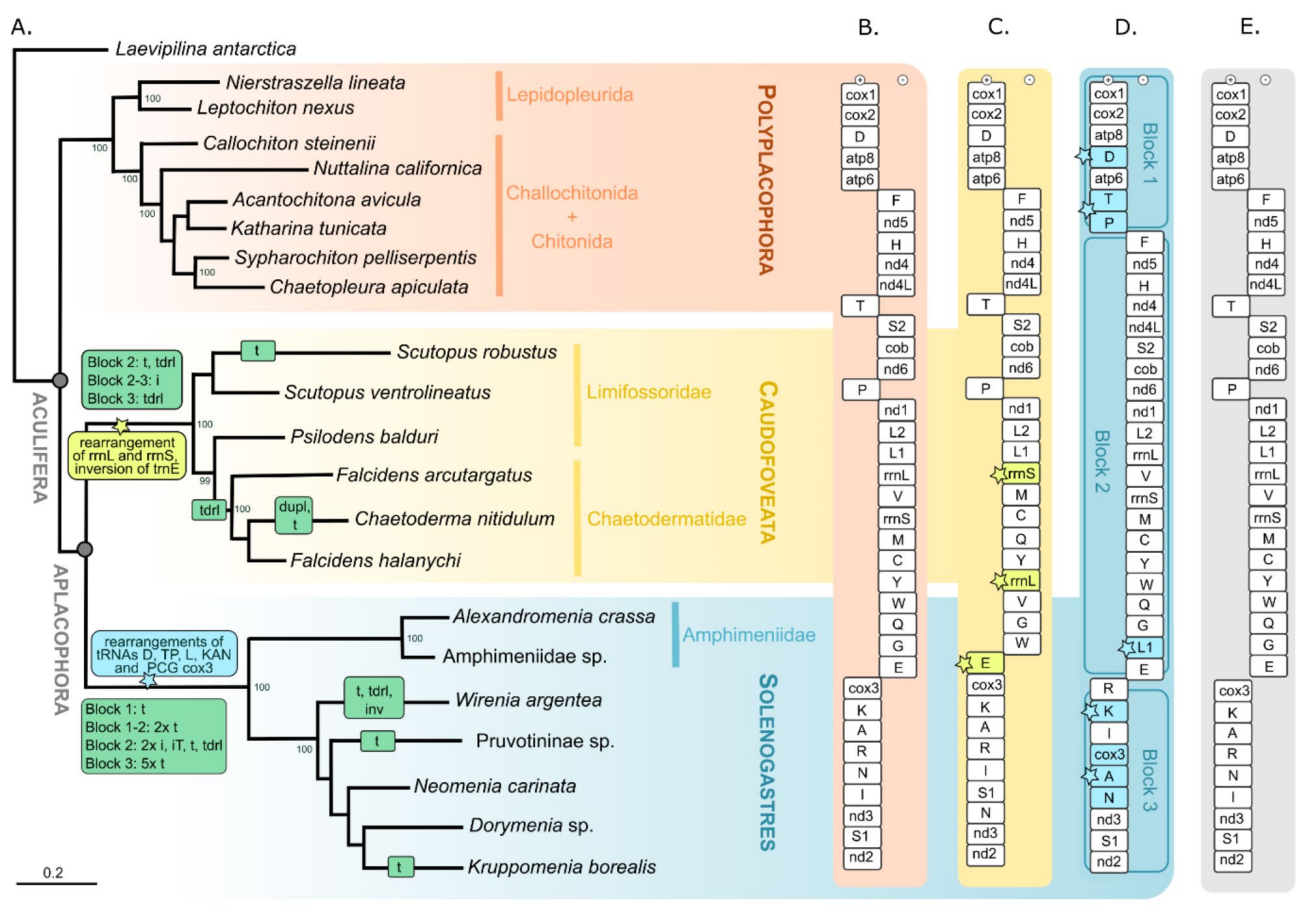
Evolution of mitochondrial gene arrangement in Aculifera [(Solenogastres + Caudofoveata) + Polyplacophora] with hypothetical ancestral gene orders. (**A**) ML phylogeny of Aculifera based on 13 protein coding genes translated into amino acids. BS values of 99 or higher shown. Green boxes: hypothetical gene rearrangements (for number of all events see Supplementary Table 7). Blue and yellow box: hypothetical synapomorphic events regarding mitochondrial gene order. **(B)** Ancestral gene order of Polyplacophora (after [21]). **(C)** Ancestral gene order of Caudofoveata and **(D)** Solenogastres inferred through CREx analyses. **(E)** Hypothetical ancestral gene order of Mollusca (after [54]). Abbreviations: dupl, duplication, i, inversion, iT, transposition with inversion, t, transposition, tdrl, tandem duplication random loss. Preceding number indicates number of events

All Solenogastres exhibit a highly conserved gene order arranged into three main blocks (see Fig. 3). The first block on the forward strand consists of cox1, cox2, atp8, D, atp6, T, P. The second block on the reverse strand includes F, nad5, H, nad4, nad4L, S2, cob, nad6, nad1, L2, rrnL (16S rRNA), V, rrnS (12S rRNA), M, C, Y, W, Q, G, L1, E (in *Wirenia argentea* transpositioned to E, L1). The third block on the forward strand contains cox3, nad3, and nad2, along with the tRNA K, A, R, N, I, and S1 genes. However, this third block displays the most rearrangements among the investigated solenogaster mitogenomes, as the positions of the tRNA A, I, K, R, N genes are highly variable among species (Fig. 2).

Five different start codons with varying frequencies initiate transcription of protein coding genes in the studied mitogenomes (see Supplementary Table 1). The most common start codons, ATG (in total occurring 48 times across all investigated mitogenomes), ATA (occurring 27x), and ATT (11x) are found in most mitogenomes, except for *Kruppomenia borealis*, which lacks ATT. These start codons are typically present in genes such as nad1, nad4, nad5, nad6, and cob. Additional start codons TTG (2x) and GTG (2x) are present in atp6 of Pruvotininae sp., *Kruppomenia borealis*, and Amphimeniidae sp., and in nad4L of Pruvotininae sp. PCGs are mostly terminated via complete stop codons TAA (50x), TAG (15x), but also the truncated stop codons T(AA) (18x) and rarely TA(A) (once in *W. argentea*, *K. borealis*, and *N. carinata* each) which are completed post-transcriptionally through polyadenylation [9]. All investigated mitogenomes contain two types of stop codons (TAA and its truncated versions T(AA) and TA(A), as well as TAG) (see Supplementary Table 1), except for Pruvotininae sp. with only TAA (or its truncated version) as a stop codon (see Supplementary Fig. 1). The most frequently used amino acids within the PCGs of all investigated mitogenomes are Serine (8.3 – 10.9%) and Leucine (13.6 – 14.6%, see Supplementary Fig. 1 and Supplementary Table 3). - The number of intergenic non-coding regions (NCRs) with more than 10 bp within the complete mitogenomes varies between six NCRs in Pruvotininae sp. and 11 NCRs in *Dorymenia* sp. (see Supplementary Table 4). Some mitogenomes have five or fewer NCRs exceeding 100 bp in length. In Pruvotininae sp., there are 14 NCRs totaling 1,171 bp, which represents 7.63% of the mitogenome. These NCRs range in length from 1 to 586 bp. The third largest NCR (231 bp), located between trnE and trnK, has the highest AT content (86.6%) and contains a repetitive sequence of 19 bp (referred to as Motif 1, see Table 2), which could represent the origin of replication. Repetitive motifs were identified in two additional complete mitogenomes. *Dorymenia* sp. (25 NCRs, 589 bp in total, 3.45% of the total mitogenome) and *Kruppomenia borealis* (18 NCRs, 1354 bp, 8.54%) possess motifs of 19 bp and 10 bp in their NCRs between trnE and R (see Table 2 and Supplementary Table 4). Within the incomplete mitogenome of Amphimeniidae sp. (18 NCRs, 317 bp, ) we identified a short repetitive sequence towards the end of one of the contigs (Motif 4, see Table 2).

**Table 2:** Repetitive motifs found in the non-coding regions of the investigated mitogenomes

Motif No.	Taxon	NCR border	Number of repetitions of motif sequence	Length of motif
1	Pruvotininae sp.	trnE - trnK	8x CTATTATATATATATATTA	19 bp
2	*Dorymenia* sp.	trnE - trnR	4x GTTATATATATATATATAT	19 bp
3	*Kruppomenia borealis*	trnE - trnR	2x + 25x GTATATATAT	10 bp
4	Amphimeniidae sp.	cox2 – end of contig	6x ATAATTTAAATAT	13 bp

While gene overlaps are present in all investigated mitogenomes, mostly occurring between tRNA genes of the MCYWQGL1E cluster and ranging from 1 to 11 bp, the number and total length of overlapping regions vary considerably (see Supplementary Table 5). *Kruppomenia borealis* has the smallest overall gene overlap, with only a single nucleotide overlapping between cox1 and cox2. Number and length of gene overlaps in both incomplete mitogenomes of Amphimeniidae are low, with only one overlapping region of 4 bp in Amphimeniidae sp. and two overlaps of 27 bp and 31 bp in *A. crassa* due to the position of trnA on the reverse strand opposite to trnR and K (see Fig. 2, Supplementary Table 5). *Dorymenia* sp. and *W. argentea* have a total of five gene overlaps (a total of 15 bp and 28 bp, respectively). The mitogenome of Pruvotininae sp. exhibits the highest number of gene overlaps, with eight overlapping regions totaling 108 bp, including the longest overlap of 80 bp between nad6 and cob.

In general, most tRNA exhibit the typical clover-leaf structure (Supplementary Table 6) with most variation occurring in the D-loop, which is missing in trnA of Pruvotininae, *K. borealis*,* N. carinata*, and in trnR and trnQ of *Dorymenia* respectively. It is also absent in all trnL1, except in *N. carinata* which instead lacks the T-arm. Both serine tRNA genes (trnS1 and trnS2) lack the D-loop in all investigated species (see Supplementary Table 6), as reported from other molluscs [51–53].

### Phylogenetic analyses and ancestral aculiferan gene arrangements

The retrieved phylogenetic hypothesis on Aculifera based on 13 PCGs translated to amino acids (4,042 amino acids in raw alignment, 2,400 amino acids in final gblocked alignment) is shown in Fig. 3A: Aplacophora (Solenogastres + Caudofoveata) and all three classes of Aculifera are monophyletic with high bootstrap support (BS). Amphimeniidae forms the sister clade to all remaining Solenogastres. In Caudofoveata, Limifossoridae and Chaetodermatidae are monophyletic, however the chaetodermatid genus *Falcidens* is paraphyletic. In Polyplacophora, Lepidopleurida forms the sister clade to Callochitonidae + Chitonida. Maximum-likelihood analyses based on the nucleotide-dataset of 22 taxa (15,364 bp raw sequence alignment) produced an identical topology of monophyletic Solenogastres and Caudofoveata. Among monophyletic Polyplacophora, Acanthochitonina is the monophyletic sister-clade to Chitonina. But analyses based on the raw and gblocked nucleotide alignments (8,364 bp after applying Gblocks to remove ambiguously aligned sites) both show no resolution for the deep nodes and do not support Aplacophora, but rather group Caudofoveata sister to Polyplacophora (trees not shown).

All Solenogastres share the same gene arrangements and orientation regarding the PCGs and ribosomal RNAs, (Fig. 2). The most parsimonious scenario based on CREx analyses of the Aculifera mitogenomes (see supplementary Table 7) suggests that along the solenogaster stem line a transposition of the trnD gene occurred within block 1. Moreover, a tandem-duplication-random-loss (tdrl – resulting in trnT and P genes adjacent to atp6) took place between blocks 1 and 2, while on block 2 (reverse strand) trnL1 was transpositioned (see Fig. 3A). The tRNA- cluster ‘KARNI’ present in the hypothetical ancestral polyplacophoran mitogenome (Fig. 3B) and likely plesiomorphic for Aculifera (see Discussion below) is modified and dissolved independently in both classes of aplacophoran molluscs: In Solenogastres a transposition of trnK followed by a tdrl of cox3, A and N results in the hypothetical ancestral arrangement for block 3 in Solenogastres (R, K, I, cox3, A, N, nad3, S1, nad2, see Fig. 3D). In Caudofoveata a reversal of trnE and a tdrl event involving trnN and nad3 hypothetically leads to the novel order: E, cox3, K, A, R, I, S1, N, nad3, nad2 (Fig. 3C). Further, block 2 was rearranged on the Caudofoveata stem line by a transposition of tRNAs Y and W and a tdrl, which presents the most parsimonious scenario of the caudofoveate gene arrangement (rrnS, M, C, Q, Y, rrnL, V, G, W), switching the order of the two rRNA genes and rearranging the tRNA cluster ‘MCYWQGE’ present in Polyplacophora and Solenogastres.

Within Solenogastres considerable rearrangements of tRNAs most likely result from multiple reversal events based on CREx analyses. *Wirenia argentea* shows a unique transposition of trnL1 gene on block 2 and expands it by the tRNAs otherwise found in block 3 by a series of three independent inversion events, involving (1) R, K, I, (2) N, A and (3) S1, and one tdrl involving cox3 and nad3. Pruvotininae sp. shows two transpositions (of the cluster rrnS, M, C, Y, W, Q, G, L1, E and of trnI genes). *Kruppomenia borealis* a transposition of trnN gene.

Within Caudovofeata, *Scutopus robustus* shows a transposition of trnS2. Within Chaetodermatidae, along the stem line of *Falcidens* and *Chaetoderma* a tdrl occurred involving tRNAs F, G and E, cox3, K (Fig. 3A). *Chaetoderma nitidulum* uniquely shows a duplication of cox2 and two transpositions resulting in a changed order regarding trnW and tRNAs F, G.

## Discussion

### Mitochondrial genomes of Solenogastres

Solenogastres was the last class of Mollusca lacking data on a complete mitochondrial genome. Through sequencing and annotation of six mitogenomes across different taxa this study has filled a considerable gap in current knowledge on Molluscan mitogenomes. The lengths of the investigated mitogenomes range from 15,103 to 17,030 base pairs, surpassing the maximum mitogenomic sizes observed in other molluscan classes such as Scaphopoda (maximum of 14,519 bp [29]) and Polyplacophora (16,572 bp, see [21]). The largest molluscan mitogenomes are found in bivalves (approximately 56 kb) and gastropods (approximately 27 kb), typically due to the expansion of large noncoding regions (LNCRs) [9, 55]. Among the investigated Solenogastres, *Wirenia argentea* exhibits the longest mitogenome, attributed to a 1,259 bp long non coding region between trnF and trnP at the transition of the forward and reverse strand. In molluscan mitogenomes, the largest NCR typically contains the control region with the putative origin of replication. These regions are characterized by high AT contents and the presence of repetitive palindromic motifs [9, 18, 19, 56]. Consistent with this pattern, we identified unique repetitive motifs in the LNCRs of three investigated complete solenogaster mitogenomes (Pruvotininae sp., *Dorymenia* sp., *W. argentea*). All three potential origins of replication are located at the transition between the plus and minus strands, indicating a bidirectional origin of replication for both strands [57, 58]. In contrast to other aculiferan mitogenomes of Polyplacophora and Caudofoveata [21, 22], the mitochondrial genomes of Solenogastres exhibit high diversity in gene arrangements, generally resulting from tRNA transpositions and rearrangements based on the ‘duplication-random loss model’ [10].

### Exploring the potential of mitogenomics to understand the evolution of Solenogastres

Highly rearranged gene order in mitochondrial genomes can reflect compositional strand bias and high evolutionary rates, which can hamper the inference of phylogenetic hypotheses [18]. Conversely, mitochondrial genomes that evolve at slower rates and exhibit fewer rearrangements potentially result in better resolved phylogenies [21, 59]. In Polyplacophora, the presence of PCGs and rRNAs encoded on the same strand across different lineages has been proposed to reduce problematic phylogenetic inference arising from increased evolutionary rates and strand bias [21]. Although herein investigated solenogaster mitogenomes display less conserved gene arrangements compared to Polyplacophora and Caudofoveata [21, 22, 28], PCG and rRNA gene synteny is observed across the taxa examined in this study. Furthermore, the obtained topology in our phylogenetic trees is largely concordant with phylogenomic analysis [44] and broader-sampled analysis utilizing dual barcoding markers [43]: Amphimeniidae are retrieved as the sister group to all other Solenogastres, with gymnomeniid *Wirenia argentea* as the second off-shoot, and a sister group relationship between simrothiellid *Kruppomenia borealis* and proneomeniid *Dorymenia* sp. The rather weak support for some splits (see Fig. 2) likely results from the limited taxon sampling comprising representatives of six out of 23 known families, and should improve with an increased taxon sampling as shown in other molluscs [60].

### Aculiferan ancestral gene arrangements

Solenogaster mitogenomes exhibit a highly conserved gene order (concerning PCGs and rRNAs) arranged into three main blocks, which corresponds to the general arrangement observed also in Polyplacophora [21, 54]. Caudofoveata mitogenomes are also arranged in these three blocks consistent in gene order with other Aculifera, but uniquely exhibit a switched order of rrnS and rrnL. This feature also deviates from the hypothetical ancestral gene orders in conchiferan classes Monoplacophora [19], Gastropoda [59] and Cephalopoda [23] and is thus considered a synapomorphy for the class Caudofoveata [22]. In general, available data on aculiferan mitogenomes largely aligns with the hypothesized ancestral gene order for Mollusca [19, 23, 28] and shows few mitogenomic gene rearrangements concerning the PCGs and rRNAs within the evolution of each class [21, 22, 28, 54]. This contrasts with the diversity of gene arrangements in Conchifera: While the hypothesized ancestral gene order in gastropods is largely congruent with the hypothesized ancestral molluscan gene arrangement (see [59]), numerous rearrangements occur within the different gastropod clades dissolving the three main blocks [24, 61–63]. The same holds true for cephalopods with only some Octopodiformes conserving the ancestral state [23], as well as in hyper-diverse bivalve mitogenomes (see e.g [58]). So far, the available data on Scaphopoda does not reveal typical ancestral molluscan features and more data is needed to investigate the mitochondrial gene arrangements in this class and place them in the contexts of molluscan mitogenome evolution [25, 26, 29].

While the general gene order in Aculifera is highly conserved (compare to [19, 20]), considerable variation occurs concerning the position of the tRNAs: Putative synapomorphies for Solenogastres are observed in block 1, with (1) the transposition of tRNA D, which is typically found between cox2 and atp6 in Polyplacophora and Caudofoveata [21, 22], but in Solenogastres it is located between atp8 and atp6 (a gene stretch which is usually highly conserved among metazoan phyla [4]), and (2) the different position of tRNAs T and P in Solenogastres, adjacent to atp6 on the forward strand. This arrangement is most parsimoniously explained by a “tandem duplication random loss” (tdrl) according to the CREx analyses. In block 2 on the reverse strand, we find (3) a shared transposition of tnL1, which is not encoded between L2 and rrnL. This expands the ‘MCYWQGE’-cluster - present in Polyplacophora (but inversed in some taxa) [54] and a potential synapomorphy for Mollusca [20] - in Solenogastres to a ‘MCYWQGL1E’-cluster. The expansion of this cluster in the solenogaster *Wirenia argentea* via a transposition and tandem-duplication-random loss according to CREx, has resulted in a tRNA cluster of 14 tRNAs, thereby potentially representing one of the longest currently known tRNA clusters in Mollusca (e.g. compare to the ultralong mitogenome of *Placopecten magellanicus* with a cluster of 13 tRNAs in [55]). The rearrangement of the two rRNA genes in Caudofoveata also modifies this characteristic molluscan tRNA-cluster in these aplacophoran molluscs via transposition of tRNAs and a division into ‘MCQY’ and ‘VGWE’ (see Fig. 3).

Based on the hypothesis on the plesiomorphic molluscan gene arrangement of block 3 (cox3, K, A, R, N, I, nad3, S1, nad2) which is found across different molluscan classes including Polyplacophora [21, 54], we hypothesize that the transposition of trnK followed by a tdrl of cox3, tRNAs A and N results in a putative plesiomorphic pattern for Solenogastres. However, the position of the tRNAs belonging to the ‘KARNI’-cluster is highly variable among the investigated Solenogastres and more data is needed to confirm this putative ancestral order within block 3. This ‘KARNI’-cluster is likely plesiomorphic for Aculifera, but already modified to ‘KARIS1N’ in Caudofoveata based on available data [22].

trnP is located on the forward strand in Aculifera (between nad6 and nad2 on the reverse strand in Caudofoveata and Polyplacophora and at the end of block 1 in Solenogastres), while in the hypothetical ancestral state of Monoplacophora, Cephalopoda and Gastropoda it is orientated in the reverse direction between nad6 and nad4 [21, 23, 59]. However numerous reversals of tRNA P to the forward strand are reported across conchiferan taxa (e.g., in Scaphopods and Gastropoda [25, 27, 29, 64]). To evaluate whether the reverse orientation of tRNA P in the conchiferan ancestral genome or the forward orientation in the hypothetical ancestral aculiferan mitogenome presents the ancestral molluscan order, other lophotrochozoan groups might provide insights: In Annelida mitochondrial genes are generally transcribed from only one strand, except for Owenidae and Magelonidae, which bear tRNAs T and P on the reverse strand [65]. A similar condition is also present in the nemertean *Lineus viridis* [66]. While the mitochondrial genes of Brachiopoda are generally all encoded on one strand as well [67, 68], Phoronida and Entoprocta present a general three block arrangement, distributing mitochondrial genes on both strands, also showing tRNAs T and P in forward direction [67, 69], as in the hypothetical gene order of Aculifera. Thus, the reversal of trnP might present a synapomorphy for conchiferan molluscs with its orientation on the plus strand being the plesiomorphic state for Mollusca.

## Conclusion

Solenogastres was the last class of the phylum Mollusca still lacking a complete and annotated mitochondrial genome. For this study we sequenced six mitogenomes and comparatively analyzed mitogenomic architecture. Based on maximum likelihood analyses of Aculifera ((Solenogastres + Caudofoveata) + Polyplacophora)), we reconstructed hypothetical aculiferan ancestral mitogenomes and identified putative synapomorphies in the gene arrangements of aplacophoran Solenogastres and Caudofoveata. For phylogenetic analyses of Aculifera and deep splits within Solenogastres, nucleotide sequences of mitochondrial genomes are potentially unsuitable, but for phylogenetic analyses at lower taxonomic levels (corresponding to order and family level) this data set is highly promising. Overall, our study demonstrates, that mitochondrial genomes hold the potential to provide a compromise between time- and cost-intensive phylogenomic analyses and informatively limited sanger sequencing approaches towards resolving solenogaster phylogeny and their evolutionary history.

## Methods

### Taxon sampling and molecular lab work

We selected six Solenogastres taxa (*Alexandromenia crassa*, Amphimeniidae sp., *Dorymenia* sp., Pruvotininae sp., *Kruppomenia borealis*, *Wirenia argentea*) from the two most common (out of four) orders of Solenogastres, including polyphyletic “Cavibelonia”, to sequence their mitogenomes (for sampling details and voucher numbers, see Table 1). Molecular lab work, including DNA extraction, library preparation, and sequencing, was conducted by the authors at different institutions using various workflows and platforms. Details of the DNA extraction, library preparation protocols and putative mitogenome assembly for each sequenced mitogenome are provided in Table 3. The workflows followed either the standard protocols provided by the respective manufacturer or previously published protocols (see citations in Table 3). In the case of Pruvotininae sp. and *Dorymenia* sp., we amplified DNA using the GenomiPhi V2 DNA Amplification Kit prior to library preparation to obtain sufficient input DNA.

We created a custom BLAST library by downloading the publicly available caudofoveate mitogenomes of [22], the polyplacophorans *Nuttalina californica* (KJ569362.1), *Cryptochiton stelleri* (KJ569363.1) and *Sypharochiton pelliserpentis* (KJ534307.1), the monoplacophoran *Vema ewingi* (KY244019.1) and the vetigastropod *Haliotis laevigata* (NC024562.1) from GenBank. We also included an unpublished dataset of solenogaster cytochrome *c* oxidase I (COI) and 16S rRNA barcodes. We performed BLAST searches using the blastn and megablast functions as implemented in Geneious Prime 2021.2.1, with an e-value cutoff of 0.01, to identify contigs with putative solenogaster mitochondrial origin.

We retrieved the mitogenome of Pruvotininae sp. in a single, continuous contigs, while the other mitogenomes assembled into two contigs, mostly representing the forward and reverse strand. All newly sequenced mitogenomes have been deposited in GenBank (see Table 4 for accession numbers). In instances where mitogenomes were obtained as two contigs, they were submitted as a unified sequence with a placeholder gap represented by 100 Ns, in accordance with GenBank’s submission guidelines for unknown gap lengths.

**Table 3:** Molecular workflows conducted in the involved institutions to generate the novel mitogenomes for this study

Taxon	Institution	DNA extraction	Library preparation	Sequencing platform	Assembly
*Alexandromenia crassa*	UA	Omega Bio-tek EZNA MicroElute Genomic DNA kit	Illumina Nextera	Illumina HiSeq 4000, 2 × 100 bp paired-end sequencing, Macrogen South Korea	MitoZ [70]
Amphimeniidae sp.	LMU Munich	CTAB + spin column [43]	Illumina Nextera Flex	Illumina MiSeq, 2 × 300 bp paired-end sequencing (v3 chemistry) Genomics Service Unit, Faculty of Biology, LMU Munich	MitoZ [70]
*Dorymenia* sp.	MPI	CTAB	Illumina Nextera	Illumina HiSeq 2500	SOAPdenovo2 (v240) [71]
Pruvotininae sp.	MPI	CTAB	Illumina Nextera	Illumina HiSeq 2500	SOAPdenovo2 (v240) [71]
*Kruppomenia borealis*	HCGS	Autogen Prep 956 Extractor *	Kapa BioSystems HyperPlus Kit (KR1145 -v3.16) ^†^	Illumina HiSeq 2500	SPAdes v3.1.1.0 [72]
*Wirenia argentea*	HCGS	Autogen Prep 956 Extractor *	Kapa BioSystems HyperPlus Kit (KR1145 -v3.16) ^†^	Illumina HiSeq 2500	SPAdes v3.1.1.0 [72]

**Table legend**: Abbreviations institutions: HCGS, Hubbard Center for Genome Studies, LMU Munich, Ludwig-Maximilians-Universität (Munich, Germany), MPI, Max-Planck Institute for Molecular Genetics (Berlin, Germany), UA, The University of Alabama (Tuscaloosa, USA). * Tissue digestion with Autogen M2, M1 buffers and Proteinase K (see [60]). † Optimized for low-input DNA

### Mitogenome annotation and analyses

All complete and partial mitochondrial genomes were submitted to the MITOS 1 (http://mitos.bioinf.uni-leipzig.de/index.py) and MITOS 2 (http://mitos2.bioinf.uni-leipzig.de/index.py) web servers for annotation using the invertebrate mitochondrial genetic code [76, 77]. Additionally, we used the ARWEN web server (http://130.235.244.92/ARWEN/) for tRNA detection [78]. Comparative analyses with tRNAscan-SE web server [79] resulted in a considerably lower number of identified tRNA genes even when the threshold/ cut off was lowered to 0.1 (default 20) and are thus not reported. We compared the annotations of both MITOS versions and ARWEN in Geneious prime 2021.2.1 and retrieved tRNA genes were considered valid if (1) identified by both approaches (MITOS and ARWEN), or (2) if only retrieved by one approach, but determined to form a clover-leave secondary structure as predicted by the respective tool (MITOS or ARWEN). We generally followed annotation suggestions of Ghiselli [9]: PCGs were edited manually, starting at the first start codon within an Open Reading Frame (ORF Finder as implemented in Geneious 2021.2.1) and terminating either at the first complete stop codon or downstream of a tRNA genes with a truncated T or TA, which will be completed into the stop codon TAA via polyadenylation during transcription. PCGs were allowed to overlap, if reading frames differ. If predicted tRNA gene lengths differed between MITOS and ARWEN, we reported the results as suggested by ARWEN. In contrast to the workflow by Ghiselli [9], we assumed that ribosomal RNA (rRNA) genes extend to the boundaries of adjacent PCGs and edited them accordingly. We conducted alignments of each PCG including all outgroup taxa (for details see below) to further check the length of the identified genes. Strand asymmetries were calculated according to [80]: AT-skew = [A - T]/[A + T] and GC-skew = [G - C]/[G + C]. Nucleotide composition and relative synonymous codon usage (RSCU) of PCGs were computed using MEGA11 [81] and visualized with ggplot2 in R-studio.

### Phylogenetic analyses

We selected a mitogenomic dataset that included publicly available aplacophoran taxa (i.e., six representatives of Caudofoveata and a total of seven Solenogastres including the mitogenomes reported in this study) and representatives of all major clades of polyplacophorans (if available). The unverified and unannotated mitogenome of the solenogaster *Epimenia babai* (GenBank accession number MT798543.1) was not included due to its unpublished status. The conchiferan monoplacophoran *Laevipilina antarctica* was chosen as outgroup (see Table 4 for complete taxon sampling and GenBank accession numbers). We excluded the prochaetodermatid *Spathoderma clenchi* (Caudofoveata, GenBank accession number MF579534), as we interpret it as a putative contamination/ chimeric sequence based on ambiguous BLAST results.

Single gene alignments from the resulting dataset of 22 taxa were constructed for each of the 13 PCGs and two rRNA genes (16S rRNA and 12S rRNA genes) using the default setting in MUSCLE [82] as implemented in Geneious Prime 2021.2.1. We checked the resulting alignments thoroughly via translation into amino acids and used Gblocks on each single gene to remove ambiguously aligned sites using standard settings [83, 84]. Single gene alignments were concatenated in Geneious. We compared the phylogenetic signal of two different datasets: 13 PCG plus two rrnS genes as nucleotide sequences and 13 PCG as amino acid sequences. We used the IQ-Tree web server (http://iqtree.cibiv.univie.ac.at*)* [85] for phylogenetic maximum-likelihood analyses [85–88] using the mtZOA + I + G4 (amino acid dataset) and GTR + F + I + G4 (nucleotide dataset) models under BIC. The best fitting model was chosen via ModelFinder [89].The monoplacophoran *Laevipilina antarctica* served as outgroup and was drawn to the root in IQtree analyses. Ultrafast bootstrapping was used to assess nodal support [87].

**Table 4:** All mitochondrial genomes used in the present study

Classification	Species	GenBank accession number	Length, number of contigs
**SOLENOGASTRES**			
“Cavibelonia” - Amphimeniidae	*Alexandromenia crassa +*	PP333953	15,748 bp, two contigs*
“Cavibelonia” - Amphimeniidae	Amphimeniidae sp. +	PP333954	15,587 bp, two contigs*
“Cavibelonia” - Proneomeniidae	*Dorymenia* sp. +	PP333956	15,103 bp, two contigs
“Cavibelonia” - Pruvotinidae	Pruvotininae sp. +	PP333952	15,347 bp, one contig
“Cavibelonia” - Simrothiellidae	*Kruppomenia borealis +*	PP333955	15,857 bp, two contigs
Pholidoskepia - Gymnomeniidae	*Wirenia argentea +*	PP333957	17,090 bp, two contigs
Neomeniamorpha - Neomeniidae	*Neomenia carinata*	MF693834.1 [22]	12,318 bp*
**CAUDOFOVEATA**			
Chaetodermatida - Chaetodermatidae	*Chaetoderma nitidulum*	EF211990.1	21,008 bp
Chaetodermatida - Chaetodermatidae	*Falcidens acutargatus*	MF568514 [22]	14,209 bp
Chaetodermatida - Chaetodermatidae	*Falcidens halanychi*	MF568515 [22]	14,508 bp
Limifossorida - Scutopodidae	*Scutopus ventrolineatus*	KC757645 [28]	14,662 bp
Limifossorida - Scutopodidae	*Scutopus robustus*	MF579533 [22]	14,515 bp
Limifossorida - Limifossoridae	*Psilodens balduri*	MF579532 [22]	14,513 bp*
**POLYPLACOPHORA**			
Chitonida - Acanthochitonidae	*Acanthochitona avicula*	NC047426 [21]	15,203 bp
Chitonida - Callochitonidae	*Callochiton steinenii*	MN864061 [21]	11,923 bp*
Chitonida - Chitonidae	*Sypharochiton pelliserpentis*	KJ534307 [73]	15,048 bp
Chitonida - Tonicellidae	*Nuttallina californica*	KJ569362 [74]	15,604 bp
Chitonida - Mopaliidae	*Cryptochiton stelleri*	KJ569363 [74]	15,082 bp
Chitonida - Mopaliidae	*Katharina tunicata*	KTU09810 [75]	15,532 bp
Chitonida - Chaetopleuridae	*Chaetopleura apiculata*	KY824658 [54]	15,108 bp
Lepidopleurida - Nierstraszellidae	*Nierstraszella lineata*	NC047421 [21]	15,765 bp
Lepidopleurida - Lepidopleuridae	*Leptochiton nexus*	NC047422 [21]	15,488 bp
**MONOPLACOPHORA**			
Tryblidiida - Neopilinidae	*Laevipilina antarctica*	NC033380 [19]	18,642 bp

**Table legend**: *only partial mitogenomes (i.e., missing PCGs or RNAs); + sequenced for the present study

### Ancestral mitogenome reconstruction

We used the Common Interval Explorer CREx [90] run via the Galaxy Europe server [91] to heuristically infer the most parsimonious hypothesis on the ancestral gene order of Solenogastres and Caudofoveata. As CREx can only analyze complete sets of mitochondrial genes, we excluded the incomplete mitogenomes of the caudofoveate *Psilodens balduri* and the solenogasters Amphimeniidae sp., *Alexandromenia crassa*, and *Neomenia carinata*. For inference of ancestral gene arrangements, it is superfluous to analyze genomes with identical architecture, thus only *Falcidens halanynchi* was included as representative of the *Falcidens* genus with identical gene arrangement. Overlap between the rrnS and trnR genes of Pruvotininae sp. were coded with the tRNA gene preceding the rrnS. We evaluate hypothetical ancestral aculiferan mitochondrial gene orders by comparing the ancestral states of Solenogastres and Caudofoveata as inferred through CREx, with published hypotheses on polyplacophoran mitogenome gene orders [21, 54] and of conchiferan molluscs (e.g [20, 23]). (see Supplementary Material 8 for gene arrangements).

## Electronic supplementary material

Below is the link to the electronic supplementary material.

Supplementary Material 1

## Data Availability

Annotated mitochondrial genomes have been deposited in GenBank with the accession numbers PP333952, PP333953, PP333954, PP333955, PP333956, PP333957.
