# Supplementary material for: Complementing aculiferan mitogenomics: comparative characterization of mitochondrial genomes of Solenogastres (Mollusca, Aplacophora)
Source: BMC Ecol Evol. 2024 Oct 18;24:128. doi: 10.1186/s12862-024-02311-5 (PMC11488289; doi:10.1186/s12862-024-02311-5)
Supplement: Supplementary file 1 — Supplementary Material 1 [file 12862_2024_2311_MOESM1_ESM.docx]

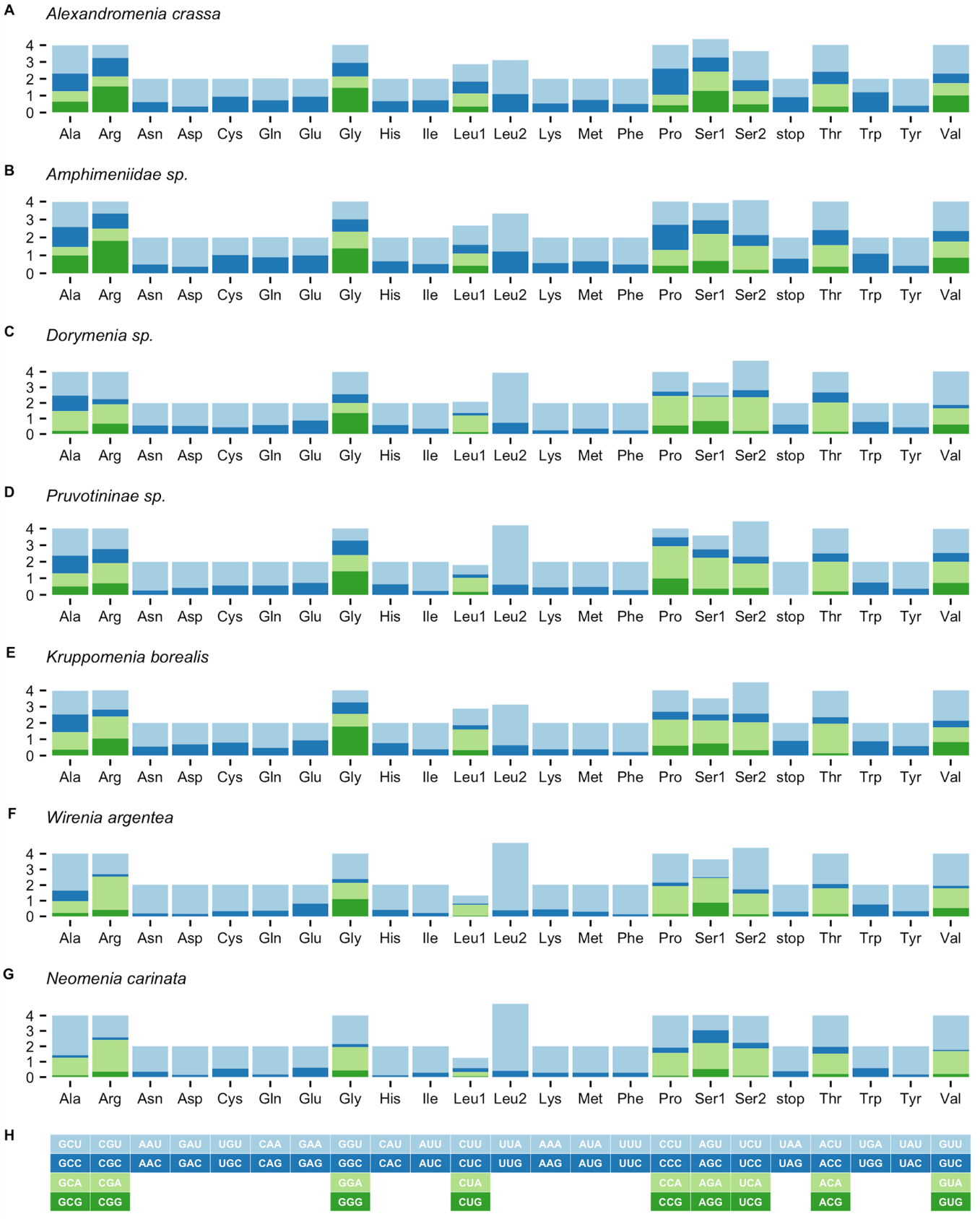


**Supplementary Figure S1.** Relative synonymous codon usage within the protein coding genes (PCGs) of investigated mitochondrial genomes (A. – G.) with color-coded respective codon families (H.)

**Supplementary Table S1. Architecture of six newly sequenced and one published solenogaster mitogenome** (+). Anticodons of tRNA: small letters in parentheses. Truncated stop codons: T.., TA.. Duplications underlined. Partial genes marked with *, missing data: x.

|  | Pruvotininae sp. | | Amphimeniidae sp. | | 1. *crassa* | | *Dorymenia* sp. | | *K. borealis* | | *W. argentea* | | *N. carinata +* | |
| --- | --- | --- | --- | --- | --- | --- | --- | --- | --- | --- | --- | --- | --- | --- |
| **Gene** | **length** | **start / stop** | **length** | **start / stop** | **length** | **start / stop** | **length** | **start / stop** | **length** | **start / stop** | **length** | **start / stop** | **length** | **start / stop** |
| **cox1** | 1503 | ATT / TAA | 1494 | ATA / TAG | 1503 | ATT / TAG | 1539 | ATG / TAA | 1536 | ATG / TAA | 1536 | ATT / TAG | 1536 | ATG / TAA |
| **cox2** | 696 | ATT / TAA | 690 | ATG / TAA | 693 | ATG / TAG | 684 | ATA / TAG | 687 | ATA / TAA | 696 | ATG / TAA | 687 | ATG / TAA |
| **atp8** | 117 | ATG / TAA | 132 | ATG / TAA | 91* | ATG / X | 117 | ATG / TAG | 124 | ATG / T.. | 117 | ATG / TAA | 117 | ATG / TAA |
| **trnD** | 63 | (gtc) | 62 | (gtc) | 62 | (gtc) | 65 | (gtc) | 67 | (gtc) | 65 | (gtc) | x | x |
| **atp6** | 699 | TTG / TAA | 694 | GTG / T.. | 699 | ATG / TAA | 699 | ATG / TAA | 709 | TTG / T.. | 696 | ATT / TAA | x | x |
| **trnT** | 61 | (tgt) | 63 | (tgt) | 68 | (tgt) | 64 | (tgt) | 65 | (tgt) | 63 | (tgt) | x | x |
| **trnP** | 62 | (tgg) | 67 | (tgg) | 67 | (tgg) | 64 | (tgg) | 65 | (ttg) | 64 | (tgg) | x | x |
| **trnF** | 63 | (ttc) | 65 | (gaa) | 64 | (gaa) | 65 | (gaa) | 66 | (gaa) | 65 | (gaa) | x | x |
| **nad5** | 1681 | ATA / T.. | 1671 | ATA / TAA | 1704 | ATG / TAG | 1680 | ATA / TAA | 1695 | ATA / TAA | 1714 | ATA / T.. | 1538* | ATA |
| **nad4** | 1314 | ATG / TAA | 1336 | ATA / T.. | 1333 | ATT / T.. | 1311 | ATT / TAA | 1336 | ATG / T.. | 1337 | ATA / TA. | 1320 | ATA / TAA |
| **trnH** | 61 | (gtg) | 63 | (gtg) | 63 | (gtg) | 65 | (gtg) | 64 | (gtg) | 63 | (gtg) | 65 | (gtg) |
| **nad4l** | 282 | GTG / TAA | 309 | ATG / TAA | 295 | ATA / T.. | 294 | ATG / TAA | 303 | ATG / TAG | 306 | ATG / TAA | 312 | ATG / TAA |
| **trnS2** | 61 | (tga) | 59 | (tga) | 61 | (tga) | 56 | (tga) | 56 | (tga) | 56 | (tga) | 57 | (tga) |
| **cob** | 1093 | ATG / T.. | 1138 | ATG / T.. | 1138 | ATG / T.. | 1074 | ATG / TAA | 1038 | ATA / TAG | 1102 | ATA / T.. | 1129 | ATG / T.. |
| **nd6** | 558 | ATA / TAA | 477 | ATT / TAA | 486 | ATC / TAA | 549 | ATG / TAA | 594 | ATA / TAA | 507 | ATG / TAA | 489 | ATA / TAA |
| **nad1** | 917 | ATA / TA. | 933 | ATG / TAA | 927 | ATG / TAG | 921 | ATG / TAA | 927 | ATG / TAA | 924 | ATG | 918 | ATA / TAA |
| **trnL2** | 67 | (taa) | 64 | (taa) | 65 | (taa) | 66 | (taa) | 70 | (taa) | 65 | (taa) | 68 | (taa) |
| **rrnL** | 1166 | - | 1322 | - | 1313 | - | 1229 | - | 1242 | - | 1233 | - | 562* | - |
| **trnV** | 64 | (tac) | 66 | (tac) | 67 | (tac) | 68 | (tac) | 63 | (tac) | 65 | (tac) | x | x |
| **rrnS** | 730 | - | 835 | - | 841 | - | 809 | - | 808 | - | 819 | - | 143* | - |
| **trnM** | 62 | (cat) | 67 | (cat) | 66 | (cat) | 68 | (cat) | 67 | (cat) | 64 | (cat) | 64 | (cat) |
| **trnC** | 63 | (gca) | 61 | (gca) | 61 | (gca) | 68 | (gca) | 64 | (gca) | 68 | (gca) | 63 | (gca) |
| **trnY** | 63 | (gta) | 63 | (gta) | 63 | (gta) | 63 | (gta) | 62 | (gta) | 62 | (gta) | 60 | (gta) |
| **trnW** | 65 | (tca) | 66 | (tca) | 65 | (tca) | 64 | (tca) | 68 | (tca) | 64 | (tca) | 63 | (tca) |
| **trnQ** | 75 | (ttg) | 69 | (ttg) | 69 | (ttg) | 66 | (ttg) | 73 | (ttg) | 76 | (ttg) | 70 | (ttg) |
| **trnG** | 65 | (tcc) | 61 | (tcc) | 61 | (tcc) | 64 | (tcc) | 62 | (tcc) | 65 | (tcc) | 63 | (tcc) |
| **trnL1** | 58 | (tag) | 68 | (tag) | 66 | (tag) | 57 | (tag) | 55 | (tag) | 56 | (tag) | 56 | (tag) |
| **trnE** | 64 | (ttc) | 63 | (ttc) | 64 | (ttc) | 67 | (ttc) | 67 | (ttc) | 65 | (tcc) | x | x |
| **trnA** | 55 | (gca) | X | X | 62 | (tgc) | 76 | (tgc) | 55 | (tgc) | 69 | (tgc) | 54 | (tgc) |
| **cox3** | 792 | ATA / TAA | 312* | X / TAA | X | X | 792 | ATG / TAG | 782 | ATG / TA. | 798 | ATG / TAA | 782 | ATA / TAG |
| **trnR** | 61 | (tct) | 65 | (tcg) | 64 | (tcg) | 64 | (tcg) | 60 | (tcg) | 63 | (tcg) | x | x |
| **trnI** | 65 | (gat) | X | X | 67 | (gat) | 61 | (gat) | 65 | (gat) | 66 | (gat) | 67 | (gat) |
| **trnK** | 65 | (ttt) | 66 | (ttt) | 66 | (ttt) | 63 | (ttt) | 62 | (ttt) | 65 | (ttt) | 63 | (ttt) |
| **trnN** | 67 | (gtt) | 64 | (gtt) | 63 | (gtt) | 72 | (gtt) | 60 | (gtt) | 61 | (gtt) | 64 | (gtt) |
| **nad3** | 354 | ATT / T.. | 355 | ATG / T.. | 346 | ATA / T.. | 352 | ATG / T.. | 354 | ATG / TAG | 354 | ATA / TAA | 354 | ATG / TAG |
| **trnS1** | 62 | (tct) | 68 | (tct) | 68 | (tct) | 66 | (tct) | 66 | (tct) | 68 | (tct) | 66 | (tct) |
| **nad2** | 963 | ATA / TAA | 984 | ATG / TAA | 984 | ATG / TAA | 966 | ATG / TAG | 975 | ATG / TAA | 993 | ATT / TAA | 993 | ATG / TAA |

**Supplementary Table S2. Strand bias of all investigated mitochondrial genomes and nucleotide composition of protein coding genes (PCGs).** Skew values given as AT / GC skew.

|  | GC content | Skew of entire mt genome | Skew of PCGs | Skew of PCGs on  forward strand | Skew of PCGs on  reverse strand | A+T % of  1^st^ codon pos. | A+T % of  2^nd^ codon pos. | A+T % of 3^rd^ codon pos. |
| --- | --- | --- | --- | --- | --- | --- | --- | --- |
| Pruvotininae sp. | 27.2% | 0.0057 / 0.0124 | -0.1649 / 0.0897 | -0.1841 / 0.1212 | -0.1486 / 0.0583 | 67.2 | 67.7 | 75.9 |
| Amphimeniidae | 28.5% | -0.0293 / 0.2437 | -0.1368 / 0.0222 | -0.1929/ 0.3301 | -0.0939 / -0.2549 | 71.6 | 73.8 | 66.4 |
| *A. crassa* | 34.8% | -0.0645 / 0.3055 | -0.1347 / 0.0011 | -0.2136 / 0.3718 | -0.0790 / -0.3040 | 65.5 | 65.7 | 62.1 |
| *K. borealis* | 31.3% | -0.1308 / 0.2352 | -0.1572 / 0.0575 | -0.3255 / 0.3196 | -0.0167 / -0.1981 | 61.5 | 64.2 | 72.6 |
| *Dorymenia* sp. | 28.9% | -0.1772 / 0.2812 | -0.1345 / 0.0489 | -0.3678 / 0.3544 | 0.0569 / -0.2717 | 64.9 | 65.1 | 77.4 |
| *W. argentea* | 23.4% | -0.0813 / 0.1562 | -0.1757 / 0.1494 | -0.2905 / 0.3271 | -0.0807 / -0.0381 | 68.6 | 67.6 | 84.6 |
| *N. carinata* | 22.7% | -0.0718 / 0.2065 | -0.1238 / 0.0511 | -0.2346 / 0.2857 | -0.0428 / -0.1797 | 76.3 | 69.8 | 84.7 |

**Supplementary Table S3. Codon usage (total numbers and relative synonymous codon usage) of protein coding genes (PCGs) of the investigated mitochondrial genomes.** Stop codons marked with *.

|  | Pruvotininae sp. | | Amphimeniidae sp. | | *A. crassa* | | *K. borealis* | | *Dorymenia* sp. | | *W. argentea* | | *N. carinata* | |
| --- | --- | --- | --- | --- | --- | --- | --- | --- | --- | --- | --- | --- | --- | --- |
| Codon | Count | RSCU | Count | RSCU | Count | RSCU | Count | RSCU | Count | RSCU | Count | RSCU | Count | RSCU |
| UUU(F) | 327 | 1.71 | 216 | 1.49 | 252 | 1.5 | 287 | 1.77 | 283 | 1.77 | 331 | 1.88 | 307 | 1.7 |
| UUC(F) | 56 | 0.29 | 73 | 0.51 | 84 | 0.5 | 37 | 0.23 | 37 | 0.23 | 22 | 0.12 | 55 | 0.3 |
| UUA(L) | 321 | 3.59 | 161 | 2.03 | 187 | 2.12 | 210 | 2.5 | 268 | 3.2 | 355 | 4.29 | 239 | 4.36 |
| UUG(L) | 55 | 0.61 | 87 | 1.09 | 108 | 1.22 | 53 | 0.63 | 61 | 0.73 | 32 | 0.39 | 22 | 0.4 |
| CUU(L) | 50 | 0.56 | 82 | 1.03 | 94 | 1.06 | 84 | 1 | 59 | 0.7 | 42 | 0.51 | 37 | 0.67 |
| CUC(L) | 19 | 0.21 | 58 | 0.73 | 44 | 0.5 | 24 | 0.29 | 16 | 0.19 | 9 | 0.11 | 14 | 0.26 |
| CUA(L) | 76 | 0.85 | 60 | 0.75 | 59 | 0.67 | 105 | 1.25 | 89 | 1.06 | 56 | 0.68 | 13 | 0.24 |
| CUG(L) | 16 | 0.18 | 29 | 0.36 | 38 | 0.43 | 29 | 0.34 | 10 | 0.12 | 3 | 0.04 | 4 | 0.07 |
| AUU(I) | 321 | 1.77 | 124 | 1.28 | 180 | 1.49 | 271 | 1.6 | 262 | 1.66 | 322 | 1.78 | 310 | 1.7 |
| AUC(I) | 41 | 0.23 | 69 | 0.72 | 61 | 0.51 | 68 | 0.4 | 54 | 0.34 | 40 | 0.22 | 55 | 0.3 |
| AUA(M) | 224 | 1.53 | 95 | 1.25 | 122 | 1.33 | 240 | 1.61 | 283 | 1.64 | 273 | 1.69 | 181 | 1.71 |
| AUG(M) | 68 | 0.47 | 57 | 0.75 | 61 | 0.67 | 59 | 0.39 | 63 | 0.36 | 50 | 0.31 | 31 | 0.29 |
| GUU(V) | 78 | 1.46 | 52 | 1.69 | 57 | 1.65 | 123 | 1.86 | 124 | 2.15 | 121 | 2.04 | 86 | 2.23 |
| GUC(V) | 29 | 0.54 | 18 | 0.59 | 20 | 0.58 | 28 | 0.42 | 13 | 0.23 | 10 | 0.17 | 3 | 0.08 |
| GUA(V) | 67 | 1.26 | 22 | 0.72 | 31 | 0.9 | 60 | 0.91 | 59 | 1.02 | 74 | 1.25 | 57 | 1.48 |
| GUG(V) | 39 | 0.73 | 31 | 1.01 | 30 | 0.87 | 54 | 0.82 | 35 | 0.61 | 32 | 0.54 | 8 | 0.21 |
| UCU(S) | 80 | 2.11 | 70 | 1.72 | 77 | 1.96 | 84 | 1.93 | 75 | 1.88 | 120 | 2.64 | 80 | 1.74 |
| UCC(S) | 17 | 0.45 | 27 | 0.66 | 24 | 0.61 | 24 | 0.55 | 19 | 0.48 | 13 | 0.29 | 17 | 0.37 |
| UCA(S) | 55 | 1.45 | 31 | 0.76 | 52 | 1.32 | 73 | 1.68 | 86 | 2.15 | 60 | 1.32 | 82 | 1.78 |
| UCG(S) | 16 | 0.42 | 20 | 0.49 | 8 | 0.2 | 15 | 0.34 | 8 | 0.2 | 6 | 0.13 | 4 | 0.09 |
| CCU(P) | 15 | 0.53 | 56 | 1.4 | 30 | 1.29 | 46 | 1.3 | 45 | 1.27 | 55 | 1.83 | 47 | 2.09 |
| CCC(P) | 15 | 0.53 | 63 | 1.58 | 33 | 1.42 | 18 | 0.51 | 10 | 0.28 | 7 | 0.23 | 8 | 0.36 |
| CCA(P) | 55 | 1.95 | 24 | 0.6 | 20 | 0.86 | 56 | 1.59 | 67 | 1.89 | 53 | 1.77 | 33 | 1.47 |
| CCG(P) | 28 | 0.99 | 17 | 0.43 | 10 | 0.43 | 21 | 0.6 | 20 | 0.56 | 5 | 0.17 | 2 | 0.09 |
| ACU(T) | 61 | 1.51 | 58 | 1.59 | 56 | 1.6 | 75 | 1.62 | 70 | 1.33 | 67 | 1.93 | 71 | 2.03 |
| ACC(T) | 20 | 0.49 | 27 | 0.74 | 29 | 0.83 | 20 | 0.43 | 34 | 0.64 | 10 | 0.29 | 16 | 0.46 |
| ACA(T) | 72 | 1.78 | 48 | 1.32 | 42 | 1.2 | 83 | 1.79 | 99 | 1.88 | 56 | 1.61 | 46 | 1.31 |
| ACG(T) | 9 | 0.22 | 13 | 0.36 | 13 | 0.37 | 7 | 0.15 | 8 | 0.15 | 6 | 0.17 | 7 | 0.2 |
| GCU(A) | 74 | 1.63 | 36 | 1.67 | 20 | 1.4 | 74 | 1.46 | 69 | 1.52 | 92 | 2.37 | 59 | 2.57 |
| GCC(A) | 50 | 1.1 | 23 | 1.07 | 16 | 1.12 | 56 | 1.1 | 46 | 1.02 | 26 | 0.67 | 4 | 0.17 |
| GCA(A) | 35 | 0.77 | 13 | 0.6 | 7 | 0.49 | 54 | 1.06 | 56 | 1.24 | 29 | 0.75 | 26 | 1.13 |
| GCG(A) | 23 | 0.51 | 14 | 0.65 | 14 | 0.98 | 19 | 0.37 | 10 | 0.22 | 8 | 0.21 | 3 | 0.13 |
| UAU(Y) | 121 | 1.64 | 166 | 1.6 | 234 | 1.57 | 94 | 1.41 | 110 | 1.55 | 124 | 1.66 | 223 | 1.81 |
| UAC(Y) | 27 | 0.36 | 41 | 0.4 | 65 | 0.43 | 39 | 0.59 | 32 | 0.45 | 25 | 0.34 | 24 | 0.19 |
| UAA(*) | 13 | 2 | 100 | 1.1 | 126 | 1.18 | 7 | 1.08 | 9 | 1.38 | 11 | 1.69 | 51 | 1.62 |
| UAG(*) | 0 | 0 | 82 | 0.9 | 87 | 0.82 | 6 | 0.92 | 4 | 0.62 | 2 | 0.31 | 12 | 0.38 |
| CAU(H) | 45 | 1.36 | 58 | 1.32 | 38 | 1.33 | 43 | 1.23 | 52 | 1.42 | 48 | 1.57 | 58 | 1.87 |
| CAC(H) | 21 | 0.64 | 30 | 0.68 | 19 | 0.67 | 27 | 0.77 | 21 | 0.58 | 13 | 0.43 | 4 | 0.13 |
| CAA(Q) | 35 | 1.43 | 51 | 1.28 | 45 | 1.13 | 38 | 1.52 | 40 | 1.43 | 36 | 1.64 | 28 | 1.81 |
| CAG(Q) | 14 | 0.57 | 29 | 0.73 | 35 | 0.88 | 12 | 0.48 | 16 | 0.57 | 8 | 0.36 | 3 | 0.19 |
| AAU(N) | 127 | 1.73 | 127 | 1.37 | 159 | 1.5 | 78 | 1.46 | 95 | 1.45 | 123 | 1.8 | 224 | 1.65 |
| AAC(N) | 20 | 0.27 | 58 | 0.63 | 53 | 0.5 | 29 | 0.54 | 36 | 0.55 | 14 | 0.2 | 47 | 0.35 |
| AAA(K) | 99 | 1.56 | 93 | 1.45 | 102 | 1.44 | 94 | 1.61 | 97 | 1.75 | 101 | 1.55 | 130 | 1.7 |
| AAG(K) | 28 | 0.44 | 35 | 0.55 | 40 | 0.56 | 23 | 0.39 | 14 | 0.25 | 29 | 0.45 | 23 | 0.3 |
| GAU(D) | 53 | 1.58 | 47 | 1.65 | 47 | 1.62 | 43 | 1.32 | 48 | 1.48 | 64 | 1.83 | 53 | 1.86 |
| GAC(D) | 14 | 0.42 | 10 | 0.35 | 11 | 0.38 | 22 | 0.68 | 17 | 0.52 | 6 | 0.17 | 4 | 0.14 |
| GAA(E) | 52 | 1.27 | 38 | 1.07 | 50 | 1.01 | 44 | 1.06 | 43 | 1.12 | 43 | 1.19 | 40 | 1.38 |
| GAG(E) | 30 | 0.73 | 33 | 0.93 | 49 | 0.99 | 39 | 0.94 | 34 | 0.88 | 29 | 0.81 | 18 | 0.62 |
| UGU(C) | 41 | 1.44 | 46 | 1.06 | 33 | 0.99 | 27 | 1.2 | 31 | 1.55 | 30 | 1.67 | 30 | 1.46 |
| UGC(C) | 16 | 0.56 | 41 | 0.94 | 34 | 1.01 | 18 | 0.8 | 9 | 0.45 | 6 | 0.33 | 11 | 0.54 |
| UGA(W) | 60 | 1.26 | 34 | 0.8 | 31 | 0.9 | 55 | 1.12 | 59 | 1.23 | 60 | 1.25 | 70 | 1.43 |
| UGG(W) | 35 | 0.74 | 51 | 1.2 | 38 | 1.1 | 43 | 0.88 | 37 | 0.77 | 36 | 0.75 | 28 | 0.57 |
| CGU(R) | 16 | 1.23 | 16 | 0.77 | 7 | 0.67 | 19 | 1.17 | 26 | 1.76 | 16 | 1.31 | 16 | 1.42 |
| CGC(R) | 11 | 0.85 | 23 | 1.11 | 9 | 0.86 | 7 | 0.43 | 5 | 0.34 | 2 | 0.16 | 2 | 0.18 |
| CGA(R) | 16 | 1.23 | 12 | 0.58 | 7 | 0.67 | 22 | 1.35 | 18 | 1.22 | 26 | 2.12 | 23 | 2.04 |
| CGG(R) | 9 | 0.69 | 32 | 1.54 | 19 | 1.81 | 17 | 1.05 | 10 | 0.68 | 5 | 0.41 | 4 | 0.36 |
| AGU(S) | 32 | 0.84 | 44 | 1.08 | 38 | 0.97 | 42 | 0.97 | 33 | 0.83 | 51 | 1.12 | 45 | 0.98 |
| AGC(S) | 19 | 0.5 | 35 | 0.86 | 30 | 0.76 | 17 | 0.39 | 4 | 0.1 | 3 | 0.07 | 38 | 0.83 |
| AGA(S) | 71 | 1.87 | 46 | 1.13 | 59 | 1.5 | 61 | 1.4 | 61 | 1.53 | 71 | 1.56 | 78 | 1.7 |
| AGG(S) | 14 | 0.37 | 52 | 1.28 | 27 | 0.69 | 32 | 0.74 | 34 | 0.85 | 40 | 0.88 | 24 | 0.52 |
| GGU(G) | 39 | 0.71 | 52 | 1.07 | 34 | 0.99 | 44 | 0.74 | 82 | 1.44 | 84 | 1.61 | 68 | 1.86 |
| GGC(G) | 49 | 0.89 | 39 | 0.8 | 24 | 0.7 | 42 | 0.71 | 33 | 0.58 | 14 | 0.27 | 7 | 0.19 |
| GGA(G) | 54 | 0.99 | 33 | 0.68 | 31 | 0.91 | 45 | 0.76 | 36 | 0.63 | 54 | 1.03 | 55 | 1.51 |
| GGG(G) | 77 | 1.41 | 71 | 1.46 | 48 | 1.4 | 106 | 1.79 | 77 | 1.35 | 57 | 1.09 | 16 | 0.44 |

**Supplementary Table S4. Position and length (in bp) of non-coding regions (NCR) within the investigated solenogaster mitogenomes.** NCR (non-coding regions) with highest AT content of each mitogenome marked with *. NCR containing repetitive motifs in bold (see also Table 4). Missing: genes missing in mitogenome.

| **NCR** | Pruvotininae sp. | Amphimeniidae sp. | *Alexandromenia crassa* | *Dorymenia* sp. | *Kruppomenia borealis* | *Wirenia argentea* | *Neomenia carinata* |
| --- | --- | --- | --- | --- | --- | --- | --- |
| nad2 – cox1 | 10 | 13 | 4 | 1 | 13 | - | - |
| cox1 – cox2 | 6 | 29 | 27 | 2 | - | 7 | - |
| cox2 – atp8 | 10 | gap | 1 | 1 | - | 6 | 5 |
| atp8 - D | 1 | 10 | gap | 1 | - | - | - |
| D – atp6 | 5 | 5 | 10 | 34 | 34 | 38 | - |
| atp6 - T | - | - | 4 | 9 | - | 5 | - |
| T – P | - | 2 | 2 | - | - | 6 | missing |
| P – F | 21 | 44 * | 162 * | 172 | 733 | 1259 | - |
| F – nad5 | - | 1 | 1 | 4 | 3 | - | - |
| nad5 – H | 586 | 30 | - | 18 | 14 | - | 15 |
| H – nad4 | 354 | - | 131 | 1 | - | - | 2 |
| nad4 – nad4l | - | - | 1 | 17 | 1 | - | 13 |
| nad4 – S2 | - | - | 13 | 7 | - | - | 2 |
| S2 – cob | - | - | - | - | 3 | - | - |
| cob – nad6 | - | 3 | 6 | 1 | 10 | 1 | 2 |
| nad6 – nad1 | - | 14 | 11 | - | 2 | 23 | 2 |
| nad1 – L2 | - | - | - | 5 | - | - | 10 |
| V - rrnS | - | - | - | - | - | 3 | - |
| M – C | 2 | 26 | 8 | 7 | 2 | 2 | missing |
| C – Y | 3 | - | - | - | - | - | - |
| Y – W | - | 1 | 1 | - | - | - | - |
| W – Q | - | - | 1 | - | - | - | - |
| Q – G | - | 1 | 4 | 3 | - | - | - |
| G – L1 | 5 | 8 | 3 | 17 | 2 | G – E: 15 | - |
| L1 – E | - | - | - | 15 | - | E – L1: 16 | missing |
| E - … | **K: 231 *** | A and start of cox3 missing | A and cox3 missing | **R: 280 *** | **R: 374 *** | L1 – I: 32 | missing |
| - | cox3 – I: 6 |  | I – R: 853 | K – I: 4 | - | I – K: 1 | K – I: 2 |
| - | A – N: 2 | cox3 – R: 122 |  | I – cox3: 53 | I – cox3: 61 | K – R: 3 | - |
| - | - | R-K: 5 | R - -K: 4 | cox3 – A: 13 | cox3 – N: 17 | R – N: 5 | I – cox3: 67 |
| - | - | - | K – N: - | A – N: 17 | N – A: 6 | N – A: 4 | A – N: 2 |
| - | - | - | - | - | A – nad3: 71 | A – S1: 30 | N – nad3: 4 |
| - | - | - | - | - | nad3 – S1: 4 | S1 – cox3: 319 * | nad3 – S1: 6 |
| N – nad3 | - | 2 | 9 | 3 | - | - | - |
| S1 – nad2: | - | 1 | 1 | 4 | 4 | - | 7 |

**Supplementary Table S5. Position and length (in bp) of gene overlaps within six newly sequenced and one published solenogaster mitogenome (+).**

| Pruvotininae sp. | | Amphimeniidae sp. | | *Alexandromenia crassa* | | *Dorymenia* sp. | | *Kruppomenia borealis* | | *Wirenia argentea* | | *Neomenia carinata +* | |
| --- | --- | --- | --- | --- | --- | --- | --- | --- | --- | --- | --- | --- | --- |
| **genes** | **length** | **genes** | **length** | **genes** | **length** | **genes** | **length** | **genes** | **length** | **genes** | **length** | **genes** | **length** |
| atp6 - trnT | 6 | nad4 – nad4l | 4 | trnR - trnA | 27 | trnS2 - cob | 2 | cox1 - cox2 | 1 | nd4 - nd4l | 11 | cox1 - cox2 | 1 |
| trnT - trnP | 8 | –- | - | trnA - trnK | 31 | trnC - trnY | 2 | - | - | trnC - trnY | 1 | trnY - trnW | 2 |
| cob - nad6 | 80 | - | - | - | - | trnY - trnW | 3 | - | - | trnY - trnW | 3 | trnW - trnQ | 3 |
| nad6 – nad1 | 1 | - | - | - | - | trnW - trnQ | 3 | - | - | trnW - trnQ | 2 | nad2 - cox1 | 23 |
| trnW - trnQ | 3 | - | - | - | - | trnR - trnK | 5 | - | - | trnQ - trnG | 11 | - | - |
| trnQ - trnG | 8 | - | - | - | - | - | - | - | - | - | - | - | - |
| trnK - cox3 | 1 | - | - | - | - | - | - | - | - | - | - | - | - |
| trnI - trnA | 1 | - | - | - | - | - | - | - | - | - | - | - | - |

**Supplementary Table S6. Secondary structures of mitochondrial tRNAs of six newly sequenced and one published solenogaster mitogenome (+), including the partial mitogenome of *Neomenia carinata*, as identified using ARWEN or MITOS 1 and 2.**

|  | Pruvotininae sp. | Amphimeniidae sp. | *A. crassa* | *K. borealis* | *Dorymenia* sp. | *W. argentea* | *N. carinata +* |
| --- | --- | --- | --- | --- | --- | --- | --- |
| trnA | 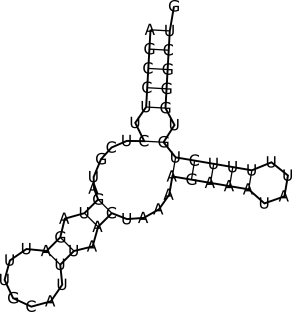 | missing | 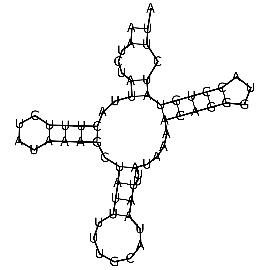 | a  g+t  g-c  t-a  t-a  t-a  a-t  a-t a  tttct  c !!!!! t  t aaaga  a t t  t t  g t  g-c  g-c  t-a  a-t  a-t  a-t  t t  t a  tgc | c  a  t-a  t-a  a-t  a-t  a-t  g+t  a-t  gg-c a  t ttct  ttgtg g !!!+ t  g tatt aagg  g :!!! a g  t ttaa t  ttaat a c  a-ttt  a-t  t-a  g-c  a-t  t t  t a  tgc | c  a  t-a  t-a  a-t  a-t  a-t  t.t  a-t  a c a  a ttc t  gta a !!! a  g tta aag t  t !! a a  t .at t  taa a a  a-taa  t-a  t-a  g-c  a-t  t t  t a  tgc | a  g-c  g-c  t+g  t-a  t-a  a-t  a-t g  ttct  t !!!! t  t aaga t  a g t  a a  t a  a-t  a-t  t-a  t-a  a-t  a-t  t t  t a  tgc |
| trnR | t  t-a  a-t  a-t  a-t  a-t  a-t  t+g a  a aat  c t !!! t  t taat tta  a !!!! a a  t atta t  a a t  t-ag  a-t  g-c  g-c  g+t  t a  c a  tct | 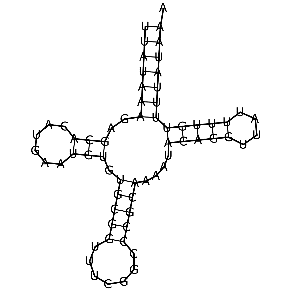 | 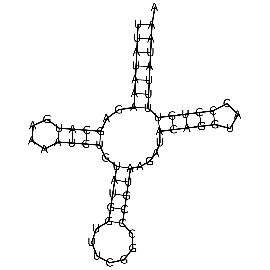 | 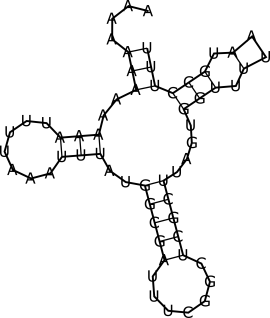 | a  t-a  t-a  g-c  t+g  g-c  c-g tt  t ccga g  t !!!! a  a ggct a  a t tt  t a  g g  t-at  c-g  a-t  g-c  a-t  t c  t g  tcg | a  t-a  a-t  t-a  t-a  t-a  a-t  a-t c  t ctct a  t a !+!! a  aacg ggga a  a !!!+ t t  ttgt a  a g g  t-aa  a-t  t-a  g-c  a-t  t c  t a  tcg | missing |
| trnN | g  t-a  a-t  g-c  a c  g-c  c-g  g-c t  a ggtaa  a a !!!!! a  t aacg ccatt a  a !!!! c a  a ttgc g  t t c  t-ata  a-t  a-t  g-c  a-t  g a  t a  gtt | 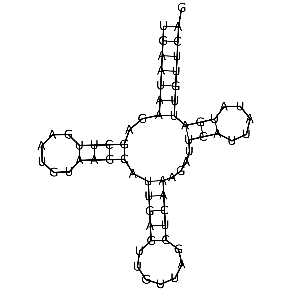 | 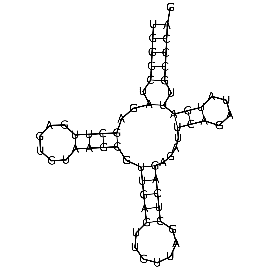 | g  t-a  a-t  a-t  a-t  t-a  a-t  g-c t  a ggtta  t a !!!!! t  a cg ccaat  a !! a a  gc t  t a t  g+tt  a-t  a-t  g-c  a-t  a a  t a  gtt | g  t-a  a-t  g+t  g-c  t-a  a-t  a-t a  a ggtat  t a !!!!! t  a cg ccata t  a !! t g  gc t  t a a  a aga  a-t  a-t  g-c  a-t  a a  t a  gtt | g  t-a  a-t  a-t  a-t  t-a  a-t  g-c a  a ggg a  t a !!! a  ttg ccc t  a !!! a aa  aac t  a a t  a-taa  a-t  a-t  g-c  a-t  a a  t a  gtt | g  t-a  a-t  a-t  a-t  t-a  a-t  g-c cat  a aggt a  a a !!!! t  a cg tcca t  a !! a aa  gc t  t a t  a-tta  a-t  a-t  g-c  a-t  a a  t a  gtt |
| trnD | t  a-t  t-a  a-t  g-c  a-t  t.t  t-a t  t gaaa  a a !!!! t  a attg cttt  t !!!! t g  a taac t  a a t  t-aa  t-a  a-t  a-t  a-t  a a  t a  gtc | 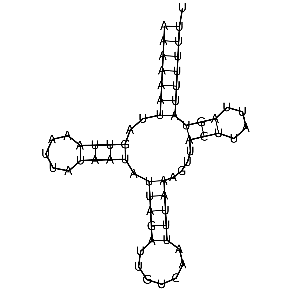 | 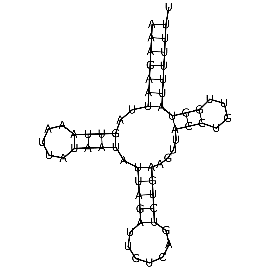 | a  a-t  t-a  t-a  a-t  g+t  a-t  t-a  c-g t  t atat  aa a !!!! a  a attg tata t  a !!!! t g  a taac t  a a a  t-aa  t-a  a-t  a-t  a-t  t g  t a  gtc | c  t-a  t-a  a-t  g-c  a-t  t.t  t-a a  t atat  aa a !!!! a  t attg tata a  a !!!+ t t  g taat t  a a g  t-aa  t-a  a-t  g-c  a-t  t t  t a  gtc | a  a-t  t-a  t-a  a-t  g-c  a-t  t-a  t.t t  t aact.  a a !!!! t  t attg ttgat  a !!!! a t  t taac t  a a g  t-aa  t-a  a-t  g-c  a-t  t a  t a  gtc | missing |
| trnC | t  a-t  a-t  t-a  c-g  t-a  a-t  g+t a  t taat  a a ! !! a  t attg aata t  a !!!! t a  t taac a  a a a  t-a  t-a  a-t  a-t  a-t  t a  t a  gca | 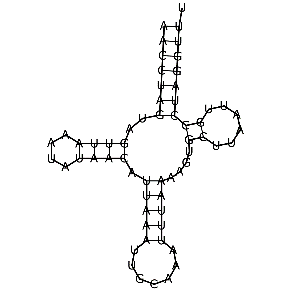 | 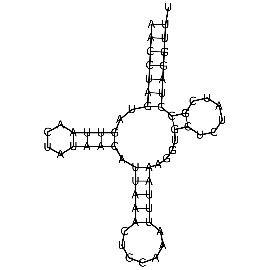 | a  a-t  a-t  a-t  t+g  c-g  c-g  t-a  g+t t  t tag  ta a !!! a  a attg atc  c !!!! a t  c taac t  a a a  t-aa  t-a  g-c  a-t  a-t  t a  t a  gca | c  t  a-t  g-c  t-a  t-a  a-t  t-a  a-t c  t caa a  ata a !!! a  a attg gtt a  a !!!! a t  t taac t  ata g a  t-aa  t-a  a-t  a-t  a-t  c a  t a  gca | a  a-t  a-t  a-t  t-a  t-a  a-t  a-t  g-c t  t cga  ata a !!! t  t ttta gct t  t !!!! a a  a aaat a  ata a a  t-aa  t-a  a-t  a-t  a-t  c a  t a  gca | 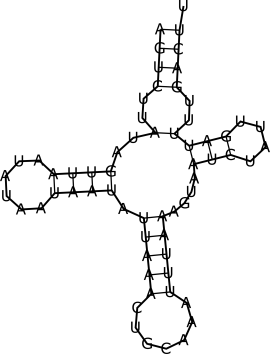 |
| trnE | 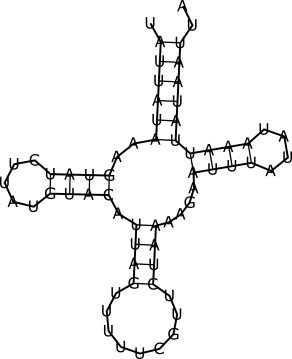 | 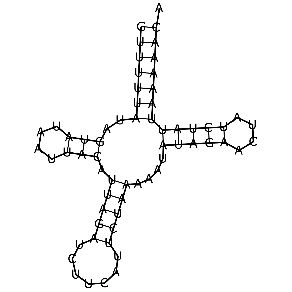 | 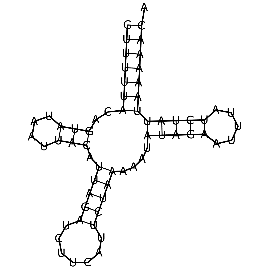 | t  t-a  a-t  a-t  t-a  t-a  t-a  t-a  a-t t  t taaat  a a !!!!! a  a tatg attta t  a !!!! t c  c atac a  a g a  c at  t-a  a-t  g-c  t-a  t t  t a  ttc | c  a  a-t  a-t  t-a  t-a  t-a  t-a  a-t a  t taatg  a a !!!!! t  a tatg attac c  a !!!! c t  a atac a  a a a  c at  t-a  a-t  a-t  c-g  t t  t g  ttc | t  a-t  t.t  t-a  c-g  t-a  t-a  a-t t  t taaa  at a !!!! t  a atg attt a  a !!+ t a  t tat a  tat a g  t-aa  t-a  a-t  g-c  a-t  t t  t a  ttc | missing |
| trnQ | a  a-t  a-t  a-t  t-a  a-t  t.t  t.t  ta-t tc  t tacc a  tata a :!!! g  g ttat ttgg c  a :!!! a tt  g tata t  gca a a  a-ta  a-t  g-c  a-t  t.t  t a  t a  ttg | 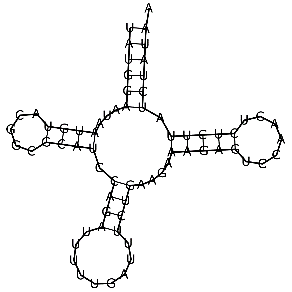 | 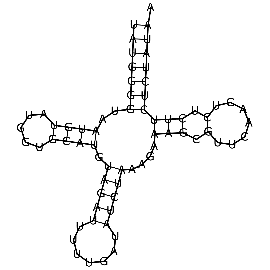 | g  t-a  t-a  t.t  t.t  a-t  t-a  a-t  ta-t ctc  t gttc a  g a +!+! g  c tatc tagg c  g !!!! a tt  g atag a  c c t  t-att  a-t  g-c  a-t  c t  t a  t a  ttg | a  t-a  a-t  a-t  c t  t-a  t.t  t.t  t-a tc  gta ttcc a  gg !!!! g  g aagg c  c a tt  at t  att t  t-act  a-t  g-c  a-t  t+g  t g  t a  ttg | t  a-t  a-t  a-t  a.g  a-t  a-t  t-a tt  a atcc c  aata !!!! a  a ttttt tagg c  c +!!+! t tt  t gaaga c  taat g a  t-aa  t-a  a-t  g-c  a-t  a-t  t t  t a  ttg | a  t-a  t.t  t.t  a-t  t-a  a-t  a-t ta  a cttcc a  at a !!!! g  t attt taagg c  g !!!! t tt  g taaa a  ca g  t-aa  a-t  g-c  a-t  t.t  c a  t a  ttg |
| trnG | a  a-t  a-t  t-a  a-t  t-a  t-a  t-a  t-a t  t aat a  a a !!! a  a tatg tta a  t !!!! t t  c atac c  a a a  g.aa  c-g  t-a  g-c  t+g  t a  t a  tcc | 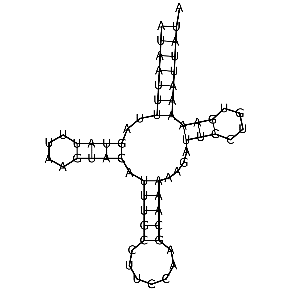 | 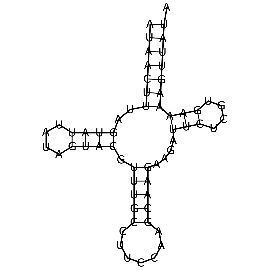 | t  a-t  t-a  a-t  t-a  t-a  t-a  t-a t  t aat  aa a !!! a  t catg tta t  t !!!+ t t  t gtat a  a a g  t+ga  t-a  t-a  g-c  t-a  t a  t a  tcc | t  a-t  a-t  t-a  a-t  t-a  c-g  t-a  t-a t  t aatt  a a !!!! t  a catt ttaa  a !!!: t a  a gtat a  a a g  g.aa  t-a  t-a  g-c  t-a  c a  t a  tcc | t  a-t  a-t  t-a  a-t  t-a  t-a  t-a  t-a t  t aat a  a a !!! c  a catg tta t  t !!!+ t t  t gtat a  a a g  t-aa  t-a  t-a  g-c  t-a  t a  t a  tcc | missing |
| trnH | a  a-t  a-t  t-a  a-t  a-t  t-a  c-g t  t tgg  a a !!! t  t tttg acc a  t !!!! t t  aaac a  a a a  t-aa  t-a  a-t  a-t  a-t  t t  t g  gtg | 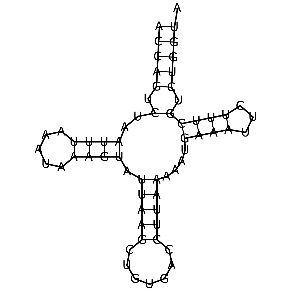 | 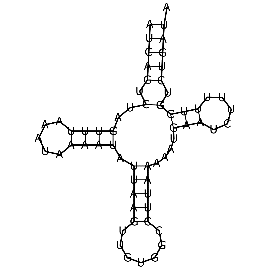 | a  a-t  a-t  t-a  t-a  a-t  a-t  a-t  c-g a  t tga  a a !!! a  a tttg act a  a !!!+ t c  t aaat a  a a g  t-aa  t-a  a-t  a-t  a-t  t t  t g  gtg | t  g-c  t-a  a a  a-t  a-t  t-a  c-g at  t tga t  a a !!! a  a tttg act a  a !!!+ t ac  a aaat a  a a g  t-aa  t-a  a-t  a-t  a-t  t t  t g  gtg | a  a-t  t-a  t-a  a-t  a-t  a-t  . g a  t agt  aa a !!! t  a tttg tca c  t !!!+ t a  a aaat a  a a g  t-aa  t-a  a-t  a-t  a-t  t g  t g  gtg | a  g-c  a-t  t-a  t-a  a-t  a-t  t-a  c-g t  t tga  aa a !!! t  a tttg act a  t !!!! t t  a aaac a  ata c a  t-aa  t-a  a-t  a-t  a-t  t a  t g  gtg |
| trnI | a  a-t  a-t  t-a  t-a  a-t  g-c  t+g  a-t t  t tttc a  a g !!!! a  gacc aaag t  a +!!! t t  ttgg a  a g a  t+gt  t-a  a-t  c-g  a-t  t t  t g  gat | missing | 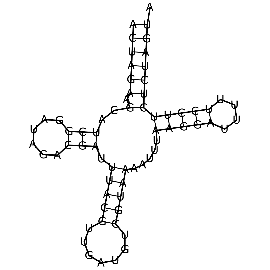 | t  t.t  t+g  t-a  a-t  a-t  t-a  g-c t  c tttcc t  g g !:!!! c  t gcc atagg t  t !!! t t  g cgg a  a g t  t-aa  t-a  a-t  c-g  g+t  t g  t g  gat | 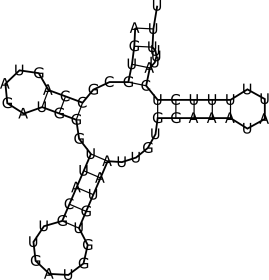 | 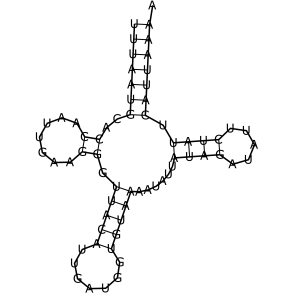 | missing |
| trnL1 | 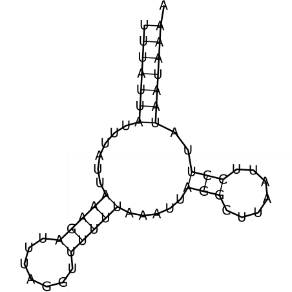 | 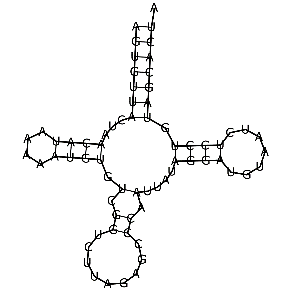 | 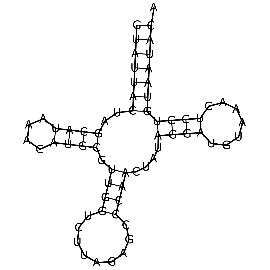 | 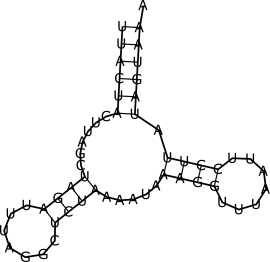 | a  t-a  t-a  a-t  c-g  t-a  a-t  c a ta  t ttcc a  a !!!! a  a aagg t  a a tt  t t  g a  a aa  t-a  a-t  g-c  t-a  c a  t a  tag | a  t-a  t-a  t-a  a-t  t-a  t-a  a-t ta  ttcc a  t !!!! a  a aagg t  a t tt  t t  c t  a-t  t-a  t-a  a-t  g-c  a-t  t a  t a  tag | a  t-a  a-t  t.t  t-a  t-a  a-t  a-tt  a g  t a g  attt a  t !!!! a  taaa a  a t a  ct-ata  t-a  a-t  g-c  a-t  t a  t a  tag |
| trnL2 | 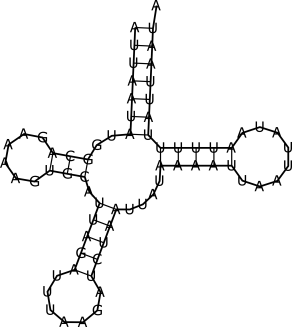 | 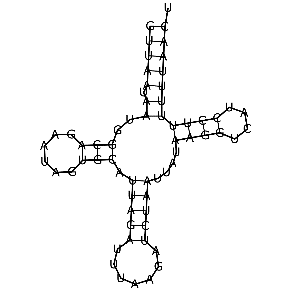 | 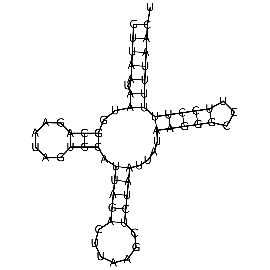 | t  t-a  t.t  t-a  t-a  a-t  a-t  t-a  a-t at  t ttttt a  ag g !!!!! t  a acg aaaaa g  a !!! t ta  a tgc a  a a c  t-aa  t-a  a-t  g-c  a-t  c a  t g  taa | a  t.t  t-a  t-a  a-t  a-t  t-a  a-t g  t ttttt a  ag g !!!!! t  t acg aaaaa a  t !!! t c  a tgc a  ag a c  t-ac  t-a  a-t  g-c  a-t  t c  t g  taa | a  t.t  t-a  t-a  a-t  a-t  t-a  a-t a  t ttctc  ag g !!!!! a  a acg aagag t  a !!! t t  a tgc a  g a t  t-at  t-a  a-t  g-c  g-c  t a  t g  taa | c  a  t.t  t-a  t-a  a-t  a-t  t-a  a-t aa  t ttttt t  ag g !!!!! t  a acg aaaaa t  a !!! t tt  a tgc a  a a t  t-at  t-a  a-t  g-c  a-t  c a  t a  taa |
| trnK | a  a-t  a-t  t+g  a-t  a-t  a-t  a-t  a-t t  t cta a  aa a !!! a  t tcg gat t  a !!! t t  a agc a  ta a a  t.tt  c-g  a-t  g-c  a-t  t a  t g  ttt | 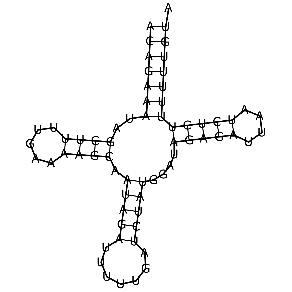 | 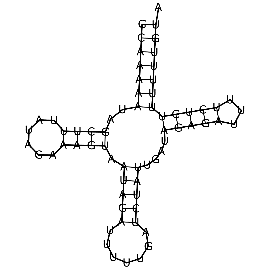 | t  g-c  c-g  a-t  a-t  a-t  a-t  a-t g  t ctt  aa a !!+ t  a tcg gag  t !!! t a  g agc a  taa g a  c-gt  c-g  a-t  g-c  c t  t g  t a  ttt | t  g-c  c-g  a-t  a-t  a-t  a-t  g-c t  g ttaat  g a !!!!! t  c tcg aatta a  t :!! t t  tgc a  a g g  g+tt  c-g  a-t  g-c  a-t  t c  t g  ttt | t  t-a  a-t  a-t  t-a  a-t  t-a  t.t  a-t t  t cttc  a a !!!! a  ttcg gaag t  a !!!! t t  aagc a  t a g  g-ca  t-a  a-t  . t  a-t  t a  t a  ttt | t  g+t  t-a  a-t  a-t  g-c  a-t  a-t t  a ctat  t a !!!! t  gtcg gata g  t +!!! t a  tagc t  t a t  a-tta  t-a  a-t  g-c  a-t  t a  t g  ttt |
| trnM | t  g+t  c-g  a-t  g-c  a-t  g-c a  t ctgt  aa a !+!! t  t tcga ggca  t !!!! t t  t agct a  a a a  t-at  t-a  g-c  g+t  g-c  c c  t a  cat | 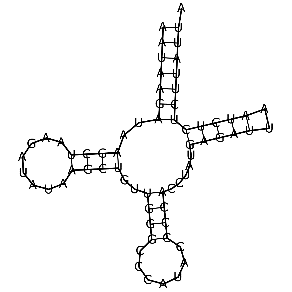 | 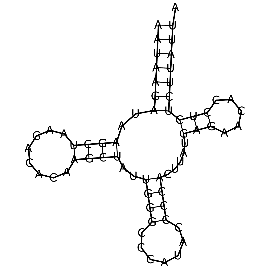 | a  a-t  a-t  t-a  a-t  a-t  g-c  a-t tt  t ctt t  aa a !!! t  t tcga gaa t  t !!!! t tt  t agct a  aa g t  t-aa  t-a  g-c  g-c  g-c  c c  t a  cat | a  a-t  g-c  t-a  a-t  a-t  a-t  a-t a  t cttat t  aa a !!!!! a  t tcga gaata t  a !!!! t t  t agct a  a a c  t-aa  t-a  g-c  g-c  g-c  c c  c a  cat | a  a-t  g+t  t-a  a-t  a-t  a-t  a-t t  t cta a  aa a !!! t  a tcga gat t  t !!!! t t  a agct a  a a t  t-at  t-a  g-c  g+t  g-c  t c  t a  cat | t  t-a  a-t  g-c  t-a  a c  a-t  a-t  a-t t  t cctt  a a !!!! t  a tcga ggaa t  t !!!! t t  t agct a  a a c  t-aa  t-a  g-c  g-c  g-c  c c  t a  cat |
| trnF | 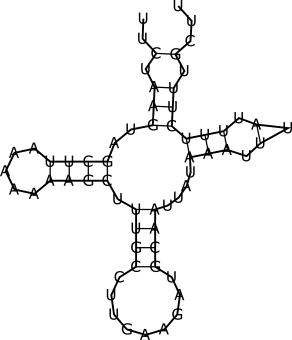 | 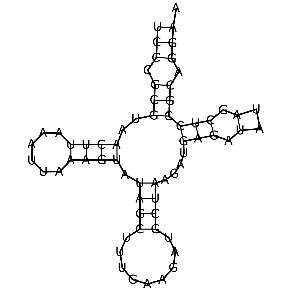 | 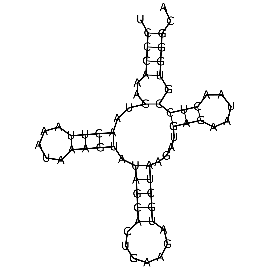 | c  c  c  t.t  t c  t+g  c-g  t-a  a-t c  t ctt a  ta a !!! a  a ctcg gaa a  c !!!! a t  c gagc g  cta a t  t-aa  a-t  a-t  c-g  a-t  c a  t g  gaa | c  c  c  t+g  t-a  t.t  t+g  c-g  t-a  a-t a  t ttat  a a !!!! t  t ttcg aata  a +!!! a c  c gagc g  a a a  t-aa  a-t  a-t  c-g  a-t  t a  t g  gaa | t  t-a  t-a  t-a  t-a  t-a  t-a  a-t  a-t g  t cttt  a a !!!! a  t ctcg gaaa  a !!!+ a t  t gagt g  a g g  t-aa  a-t  a-t  c-g  a-t  t a  t g  gaa | missing |
| trnP | c  t-a  t-a  t-a  a-t  g-c  t-a  a-t g  t cg t  ta a !! t  t ttta gc c  t !!!! t a  t aaat a  a a a  t-aa  t-a  a-t  a-t  c-g  a a  t a  tgg | 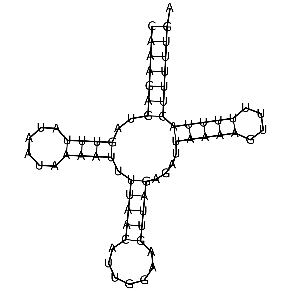 | 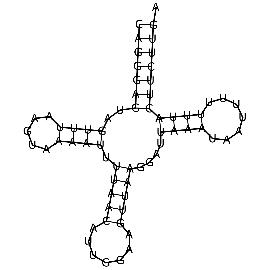 | g  t-a  t.t  t.t  a-t  g-c  t-a  a-t a  t tttcg  a a +!+!! c  t tttt gaggc  t !!!! t g  aaaa a  t t g  a-t  t-a  t-a  a-t  a-t  c-g  a a  t a  tgg | t  t-a  t+g  a-t  g-c  t.t  g-c  t-a g  a atggc  a !+!! c  t tttt aatcg c  a !!!! t  aaaa a  t t g  a-t  t-a  t-a  a-t  a-t  c-g  a a  t a  tgg | t  t.t  t-a  t+g  g+t  a-t  t-a  g-c t  t tcc t  a a !!! t  t tttt agg a  t !!!: t tt  t aaat a  a a g  t-aa  t-a  a-t  a-t  c-g  a a  t a  tgg | missing |

| trnS1 | t  a-t  a-t  a-t  g-c  a-t  t-a  t-a tat  t gttat a  ta !!+!! a  a cagta t  a g aa  aa t  t t  t-aa  a-t  a-t  a-t  g-c  c a  t a  tct | 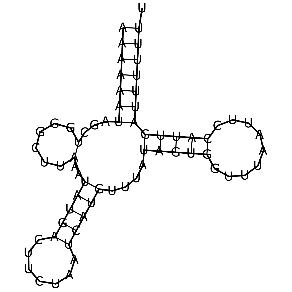 | 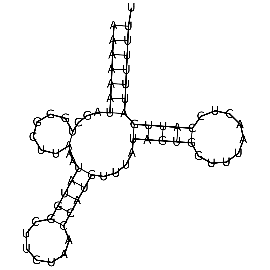 | t  a-t  a-t  a-t  a-t  g+t  t-a  c-g tc  tg attacc a  gt !!+!!! a  t tagtgg t  g a tt  ct t  aa t  c-g  t-a  g+t  g+t  a-t  g-c  c a  t a  tct | t  a-t  a-t  a-t  a-t  a-t  t+g  t-a tt  tg attacc a  gt !!+!!! a  t tagtgg a  g a tt  ct t  aa t  t-a  t-a  a-t  g+t  g-c  g-c  c a  t a  tct | t  a-t  a-t  a-t  a-t  a-t  t-a  t-a tat  ttag attac a  tt !!+!! a  t tagtg t  a a ac  tc t  taa t  t-a  t+g  a-t  a-t  a-t  g-c  c a  t a  tct | t  a-t  a-t  a-t  a-t  a-t  a a  t-a tt  t attacc a  a g !!+!!! a  ata tagtgg a  t !! a tt  taa t  c t t  t+ga  a-t  a-t  a-t  g-c  c a  t a  tct |
| --- | --- | --- | --- | --- | --- | --- | --- |
| trnS2 | g  a-t  a-t  t-a  t-a  a-t  g-c  t-a  a-t c  at tcata t  at !!!!! t  a agtat a  a a a  aa a  a t  t-ac  t-a  t-a  g-c  a-t  t c  t a  tga | 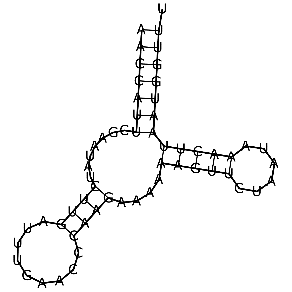 | 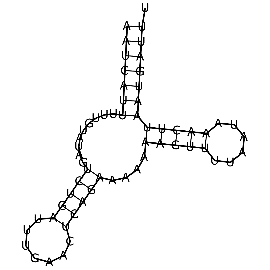 | c  t-a  t-a  t-a  a-t  a-t  a-t  t-a  t c  tg a g  a aatg g  a !!!! a  t ttac c  tta t t  t-a  t-a  t-a  g-c  g+t  c t  c g  tga | 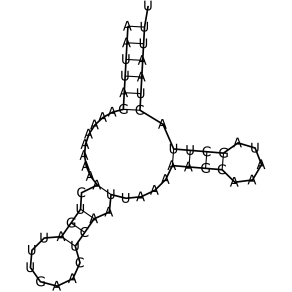 | 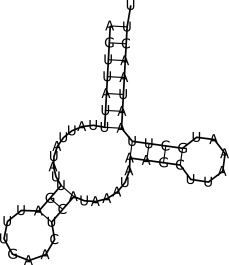 | c  a.g  t-a  t-a  t-a  a-t  t-a  a acc  a g  t a a  t aata a  a !!!! c  g ttat a  t a a  t-aaa  t-a  t-a  g-c  a-t  t c  t a  tga |
| trnT | t  t-a  a-t  c-g  t-a  a-t  a-t  aa-t a  t actat t  aa a !!!!! g  g tttt tgata t  t !!!! t t  t aaaa a  a a a  t-aa  a-t  g-c  a-t  t-a  t a  t a  tgt | 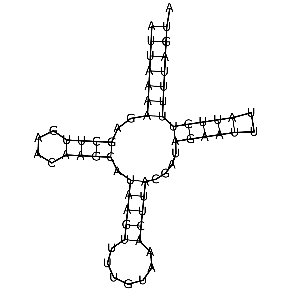 | 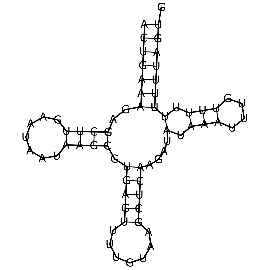 | t  t-a  a-t  c-g  t-a  a-t  g+t  a-t  a-t a  t ctat  a a !!:+ g  a ttcg gagg t  a !!!! t t  t aagc g  a g a  c-gag  a-t  g-c  a-t  t+g  g a  t a  tgt | t  g-c  t-a  t-a  a-t  a-t  g+t  a-t a  t tagt  a a +!!! t  t tttg gtca  t !!!+ g t  a aaat t  a a a  t-aag  a-t  g-c  a-t  t.t  t a  t a  tgt | g  a-t  c-g  t-a  a-t  t.t  t.t  a-t a  t ctat  aa a !!!! t  t tcg gata t  a !!! t t  t agc a  a a t  t+ga  a-t  g-c  g+t  t-a  t a  t a  tgt | missing |
| trnW | c  t-a  t-a  a-t  a-t  g-c  t+g  t-a a  t tctg  ta a !!!+ g  a ttgc agat a  t !!! a t  t aacc t  a a t  t-ac  a-t  a-t  g-c  t-a  t a  t a  tca | 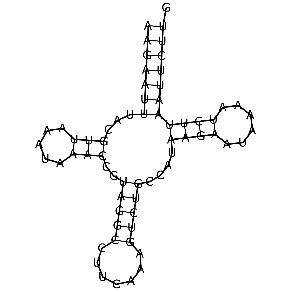 | 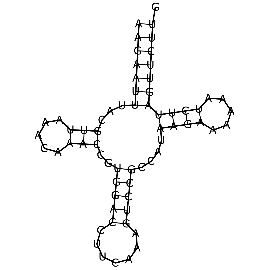 | c  t-a  t.t  t-a  a-t  a-t  g-c  tt-a a  t atctt t  a a !!!!! a  t ttgc tagaa a  a !!! a tt  t aacc a  a c a  t-a  a-t  a-t  g-c  t-a  t a  t a  tca | c  t-a  a-t  a-t  g-c  t-a  t-a a  t tctt t  a a !!!! a  t ttgc agaa t  t !!! t at  t aacc a  a t a  t-ac  a-t  a-t  g-c  t-a  t a  t a  tca | c  t-a  t.t  a-t  g-c  c-g  t-a a  t ttta a  a a !!!! t  t ttgg aaat a  a !!!! a ac  a aacc a  a a a  t-aa  a-t  a-t  g-c  t-a  t a  t a  tca | missing |
| trnY | 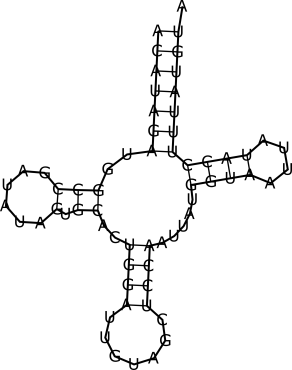 | 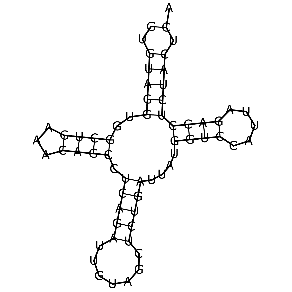 | 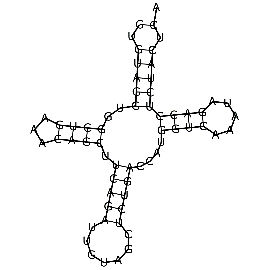 | c  a  a-t  t+g  a-t  c-g  a-t  g-c  a-t c  t ccg c  g g !!! a  a tca ggc a  a !!! t t  a agt a  a t t  t-at  t-a  g-c  g-c  g-c  t c  t g  gta | g  t-a  a-t  c-g  a-t  t-a  a-t  a-t  a-t t  t ccg c  a g !!! a  gtca ggc a  a +!!! t c  tagt a  a t t  t-at  t-a  g-c  g-c  a-t  t c  t g  gta | a  t-a  a-t  c-g  a-t  t-a  a-t  a-t  a-t t  t cca  g g !!! a  a tcg ggt a  a !!+ t t  a agt a  a a t  a aa  t-a  g-c  g-c  g-c  t c  t g  gta | missing |
| trnV | a  a-t  t-a  t-a  a-t  a-t  a-t  t-a  g-c a  t tcg  a a !!! a  a tata agc t  t !!!! t t  a atat a  a a a  t+gt  t-a  t-a  t-a  a-t  t c  t a  tac | 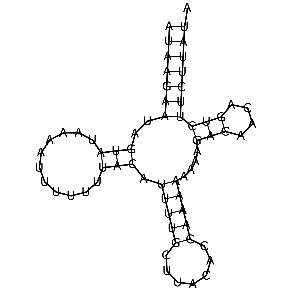 | 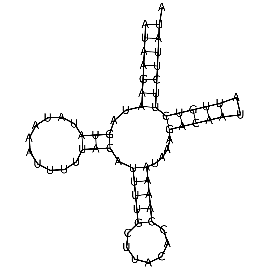 | t  t-a  a-t  a-t  a-t  a-t  c-g  g-c t  t tca a  a a !!! a  t tata agt t  t :!!! a t  t ttat a  a a a  t-aa  t-a  t-a  t-a  a-t  t c  t a  tac | a  t-a  t-a  a-t  a-t  a-t  a-t  c-g  g-c t  t ttg t  ta a !!! a  a tata aac c  t :!!! a tt  t ttat g  ta a a  t-aa  t-a  t-a  t-a  a-t  c c  t a  tac | a  a-t  a-t  a-t  a-t  a-t  t-a  g-c aa  t tc a  ta a !! g  a tata ag a  a :!!! a aa  t ttat a  ta a a  t-aa  t-a  t-a  c-g  a-t  c c  t a  tac | missing |

**Supplementary Table S7. Output of the CREx analysis to infer the ancestral mitochondrial gene orders of Aplacophora (Solenogastres and Caudofoveata), based on complete sets of all 37 mitochondrial genes.** Abbreviations: I(X), inversions of the genes listed in X, T(X , Y ,), transposition of the order of gene sets X and Y, iT(X, Y), same as transposition but with additional inversion of one of the gene sets, TDRL(X, Y), tandem duplication random loss where gene set X is kept in the first copy and gene set Y is kept in the second copy.

| Source Gene Order | Target Gene order | Rearrangement | Breakpoints |
| --- | --- | --- | --- |
| Pruvotininae sp. | *Kruppomenia borealis* | T(r ,rrns12s m c y w q g l1 e ,) | -v~r,r~-rrns12s,-e~k,k~cox3,cox3~i,i~a,a~n,n~nad3 |
|  |  | T(cox3 ,i ,) |  |
|  |  | T(a ,n ,) |  |
| Pruvotininae sp. | *Dorymenia* sp. | T(r ,rrns12s m c y w q g l1 e ,) | -v~r,r~-rrns12s,-e~k,k~cox3,cox3~i,i~a |
|  |  | T(cox3 ,i ,) |  |
| Pruvotininae sp. | *W. argentea* | T(l1 ,e ,) | -v~r,r~-rrns12s,-g~-l1,-l1~-e,-e~k,k~cox3,cox3~i,i~a,n~nad3,nad3~s1,s1~nad2 |
|  |  | TDRL(rrns12s m c y w q g l1 e k i ,r a n s1 ,) |  |
|  |  | TDRL(i a n s1 ,k cox3 nad3 ,) |  |
|  |  | I(r ) |  |
|  |  | I(k ) |  |
|  |  | I(i a n ) |  |
|  |  | I(s1 ) |  |
| Pruvotininae sp. | Caudofoveata ancestor | T(atp8 ,d ,) | cox2~atp8,atp8~d,d~atp6,atp6~t,t~p,p~-f,-nad4l~-s2,-nad6~-nad1,-l2~-rrnl16s,-v~r,r~-rrns12s,-c~-y,-y~-w,-w~-q,-q~-g,-g~-l1,-l1~-e, e~k, k~cox3,cox3~i,i~a,a~n,nad3~s1,s1~nad2 |
|  |  | T(t ,p f nad5 h nad4 nad4l ,) |  |
|  |  | T(t f nad5 h nad4 nad4l s2 cob nad6 ,p ,) |  |
|  |  | T(k ,cox3 ,) |  |
|  |  | T(n nad3 ,s1 ,) |  |
|  |  | TDRL(rrnl16s v rrns12s m c y q g l1 ,r w e k cox3 i a ,) |  |
|  |  | TDRL(r rrns12s m c w q l1 e k cox3 i a ,rrnl16s v y g ,) |  |
|  |  | TDRL(y w l1 e k cox3 a ,rrnl16s v r rrns12s m c q g i ,) |  |
|  |  | I(e ) |  |
| Pruvotininae sp. | Solenogastres ancestor | T(r ,rrns12s m c y w q g l1 e ,) | -v~r,r~-rrns12s,-e~k,k~cox3,cox3~i,i~a |
|  |  | T(cox3 ,i ,) |  |
| *K. borealis* | *Dorymenia* sp. | T(a ,n ,) | cox3~n,n~a,a~nad3 |
| *K. borealis* | *W. argentea* | T(l1 ,e ,) | -g~-l1,-l1~-e,-e~r,i~cox3,cox3~n,n~a,a~nad3,nad3~s1,s1~nad2 |
|  |  | T(a ,n ,) |  |
|  |  | TDRL(a n s1 ,cox3 nad3 ,) |  |
|  |  | I(r k i ) |  |
|  |  | I(a n ) |  |
|  |  | I(s1 ) |  |
| *K. borealis* | Caudofoveata ancestor | T(atp8 ,d ,) | cox2~atp8,atp8~d,d~atp6,atp6~t,t~p,p~-f,-nad4l~-s2,-nad6~-nad1,-l2~-rrnl16s,-v~-rrns12s,-c~-y,-y~-w,-w~-q,-q~-g,-g~-l1,-l1~-e,-e~r,r~k,k~i,i~cox3,cox3~n,n~a,a~nad3,nad3~s1,s1~nad2 |
|  |  | T(t ,p f nad5 h nad4 nad4l ,) |  |
|  |  | T(t f nad5 h nad4 nad4l s2 cob nad6 ,p ,) |  |
|  |  | T(y w ,q g ,) |  |
|  |  | TDRL(rrnl16s v g ,rrns12s m c y q ,) |  |
|  |  | TDRL(k cox3 a ,r i n s1 ,) |  |
|  |  | TDRL(cox3 i s1 ,k a n nad3 ,) |  |
|  |  | I(rrnl16s v rrns12s m c y w q g ) |  |
|  |  | I(e ) |  |
|  |  | iT(rrnl16s v rrns12s m c y w q g ,l1 ,) |  |
| *K. borealis* | Solenogastres ancestor | T(a ,n ,) | cox3~n,n~a,a~nad3 |
| *Dorymenia* sp. | *W. argentea* | T(l1 ,e ,) | -g~-l1,-l1~-e,-e~r,i~cox3,cox3~a,n~nad3,nad3~s1,s1~nad2 |
|  |  | TDRL(a n s1 ,cox3 nad3 ,) |  |
|  |  | I(r k i ) |  |
|  |  | I(a n ) |  |
|  |  | I(s1 ) |  |
| *Dorymenia* sp. | Caudofoveata ancestor | T(atp8 ,d ,) |  |
|  |  | T(t ,p f nad5 h nad4 nad4l ,) |  |
|  |  | T(t f nad5 h nad4 nad4l s2 cob nad6 ,p ,) |  |
|  |  | T(r k i ,cox3 a ,) |  |
|  |  | T(r i ,k cox3 a ,) |  |
|  |  | T(r a ,k ,) |  |
|  |  | T(y w ,q g ,) |  |
|  |  | T(k ,cox3 i ,) |  |
|  |  | T(n nad3 ,s1 ,) |  |
|  |  | TDRL(rrnl16s v g ,rrns12s m c y q ,) |  |
|  |  | I(rrnl16s v rrns12s m c y w q g ) |  |
|  |  | I(e ) |  |
|  |  | iT(rrnl16s v rrns12s m c y w q g ,l1 ,) |  |
| *Dorymenia* sp | Solenogastres ancestor |  |  |
| *W. argentea* | Pruvotininae sp. | T(r rrns12s m c y w q g l1 e k cox3 i a n nad3 ,s1 ,) | -v~-rrns12s,-g~-e,-e~-l1,-l1~-i,-i~-k,-k~-r,-r~-n,-a~-s1,-s1~cox3,cox3~nad3,nad3~nad2 |
|  |  | T(l1 ,e ,) |  |
|  |  | TDRL(r i s1 ,rrns12s m c y w q g l1 e k a n ,) |  |
|  |  | TDRL(rrns12s m c y w q g l1 e k cox3 ,i a n ,) |  |
|  |  | I(r k i a n s1 ) |  |
| *W. argentea* | Caudofoveata ancestor | T(atp8 ,d ,) | cox2~atp8,atp8~d,d~atp6,atp6~t,t~p,p~-f,-nad4l~-s2,-nad6~-nad1,-l2~-rrnl16s,-v~-rrns12s,-c~-y,-y~-w,-w~-q,-q~-g,-g~-e,-e~-l1,-l1~-i,-i~-k,-k~-r,-r~-n,-n~-a,-a~-s1,-s1~cox3,cox3~nad3 |
|  |  | T(t ,p f nad5 h nad4 nad4l ,) |  |
|  |  | T(t f nad5 h nad4 nad4l s2 cob nad6 ,p ,) |  |
|  |  | T(rrnl16s v rrns12s m c y w q g e ,l1 ,) |  |
|  |  | T(r i a n s1 ,k ,) |  |
|  |  | T(y w ,q g ,) |  |
|  |  | TDRL(rrns12s m c y q ,rrnl16s v g ,) |  |
|  |  | TDRL(k n s1 ,r i a ,) |  |
|  |  | I(e ) |  |
|  |  | iT(r k i a n s1 ,cox3 ,) |  |
| *W. argentea* | Solenogastres ancestor | T(l1 ,e ,) | -g~-e,-e~-l1,-l1~-i,-r~-n,-a~-s1,-s1~cox3,cox3~nad3,nad3~nad2 |
|  |  | T(cox3 ,a n ,) |  |
|  |  | T(cox3 a n nad3 ,s1 ,) |  |
|  |  | I(r k i ) |  |
|  |  | I(a n s1 ) |  |
| *Scutopus robustus* | *Scutopus ventrolineatus* | T(s2 ,cob ,) | t~-cob,-cob~-s2,-s2~-nad6 |
| *S. robustus* | *Falcidens acutargatus* | T(s2 ,cob ,) | atp6~-f,-f~-nad5,t~-cob,-cob~-s2,-s2~-nad6,-v~-g,-g~-w,-w~e,k~a,i~s1 |
|  |  | TDRL(t p nad5 h nad4 nad4l s2 cob nad6 nad1 l2 rrnl16s v r rrns12s m c y w q l1 i a ,f g e k cox3 ,) | |
| *S. robustus* | *Chaetoderma nitidulum* | T(t p nad4 nad4l s2 cob nad6 nad1 l2 rrnl16s v rrns12s m c y q l1 ,w g ,) | atp6~-f,-f~-nad5,-h~-nad4,t~-cob,-cob~-s2,-s2~-nad6,-v~-g,-w~e,k~a,i~s1 |
|  |  | T(f ,nad5 h ,) |  |
|  |  | T(s2 ,cob ,) |  |
|  |  | T(r i a ,e k cox3 ,) |  |
| *S. robustus* | *Falcidens halanychi* | T(s2 ,cob ,) | atp6~-f,-f~-nad5,t~-cob,-cob~-s2,-s2~-nad6,-v~-g,-g~-w,-w~e,k~a,i~s1 |
|  |  | TDRL(t p nad5 h nad4 nad4l s2 cob nad6 nad1 l2 rrnl16s v r rrns12s m c y w q l1 i a ,f g e k cox3 ,) | |
| *S. robustus* | Caudofoveata ancestor | T(s2 ,cob ,) | t~-cob,-cob~-s2,-s2~-nad6 |
| *S. robustus* | Solenogastres ancestor | T(atp8 ,d ,) | cox2~d,d~atp8,atp8~atp6,atp6~-f,-nad4l~t,t~-cob,-cob~-s2,-s2~-nad6,-nad6~p,p~-nad1,-l2~-l1,-l1~-rrns12s,-c~-q,-q~-y,-y~-rrnl16s,-v~-g,-g~-w,-w~e,e~cox3,cox3~k,k~a,a~r,r~i,i~s1,s1~n,nad3~nad2 |
|  |  | T(s2 ,cob ,) |  |
|  |  | T(rrnl16s v ,rrns12s m c y w q ,) |  |
|  |  | T(r k i ,cox3 a ,) |  |
|  |  | T(r i ,k cox3 a ,) |  |
|  |  | T(r a ,k ,) |  |
|  |  | T(k ,cox3 i ,) |  |
|  |  | T(n nad3 ,s1 ,) |  |
|  |  | TDRL(t p ,f nad5 h nad4 nad4l s2 cob nad6 ,) |  |
|  |  | TDRL(rrnl16s v q g ,y w ,) |  |
|  |  | I(rrnl16s v rrns12s m c y w q g ) |  |
|  |  | I(e ) |  |
|  |  | iT(rrnl16s v rrns12s m c y w q g ,l1 ,) |  |
| *S. ventrolineatus* | *F. acutargatus* | TDRL(t p nad5 h nad4 nad4l s2 cob nad6 nad1 l2 rrnl16s v r rrns12s m c y w q l1 i a ,f g e k cox3 ,) | atp6~-f,-f~-nad5,-v~-g,-g~-w,-w~e,k~a,i~s1 |
| *S. ventrolineatus* | *Ch nitidulum* | T(t p nad4 nad4l s2 cob nad6 nad1 l2 rrnl16s v rrns12s m c y q l1 ,w g ,) | atp6~-f,-f~-nad5,-h~-nad4,-v~-g,-w~e,k~a,i~s1 |
|  |  | T(f ,nad5 h ,) |  |
|  |  | T(r i a ,e k cox3 ,) |  |
| *S. ventrolineatus* | *F. halanychi* | TDRL(t p nad5 h nad4 nad4l s2 cob nad6 nad1 l2 rrnl16s v r rrns12s m c y w q l1 i a ,f g e k cox3 ,) | atp6~-f,-f~-nad5,-v~-g,-g~-w,-w~e,k~a,i~s1 |
| *S. ventrolineatus* | Caudofoveata ancestor |  |  |
| *S. ventrolineatus* | Solenogastres ancestor | T(atp8 ,d ,) | cox2~d,d~atp8,atp8~atp6,atp6~-f,-nad4l~t,t~-s2,-nad6~p,p~-nad1,-l2~-l1,-l1~-rrns12s,-c~-q,-q~-y,-y~-rrnl16s,-v~-g,-g~-w,-w~e,e~cox3,cox3~k,k~a,a~r,r~i,i~s1,s1~n,nad3~nad2 |
|  |  | T(rrnl16s v ,rrns12s m c y w q ,) |  |
|  |  | T(r k i ,cox3 a ,) |  |
|  |  | T(r i ,k cox3 a ,) |  |
|  |  | T(r a ,k ,) |  |
|  |  | T(k ,cox3 i ,) |  |
|  |  | T(n nad3 ,s1 ,) |  |
|  |  | TDRL(t p ,f nad5 h nad4 nad4l s2 cob nad6 ,) |  |
|  |  | TDRL(rrnl16s v q g ,y w ,) |  |
|  |  | I(rrnl16s v rrns12s m c y w q g ) |  |
|  |  | I(e ) |  |
|  |  | iT(rrnl16s v rrns12s m c y w q g ,l1 ,) |  |
| *F. acutargatus* | *S. robustus* | T(s2 ,cob ,) | atp6~-nad5,t~-s2,-s2~-cob,-cob~-nad6,-v~-w,-w~a,i~-f,-f~-g,-g~e,k~s1 |
|  |  | TDRL(t p f nad5 h nad4 nad4l s2 cob nad6 nad1 l2 rrnl16s v rrns12s m c y q g l1 ,r w e k cox3 i a ,) | |
|  |  | TDRL(f w e k cox3 ,t p nad5 h nad4 nad4l s2 cob nad6 nad1 l2 rrnl16s v r rrns12s m c y q g l1 i a ,) | |
| *F. acutargatus* | *S. ventrolineatus* | TDRL(t p f nad5 h nad4 nad4l s2 cob nad6 nad1 l2 rrnl16s v rrns12s m c y q g l1 ,r w e k cox3 i a ,) | atp6~-nad5,-v~-w,-w~a,i~-f,-f~-g,-g~e,k~s1 |
|  |  | TDRL(f w e k cox3 ,t p nad5 h nad4 nad4l s2 cob nad6 nad1 l2 rrnl16s v r rrns12s m c y q g l1 i a ,) | |
| *F. acutargatus* | *Ch. nitidulum* | T(t p nad4 nad4l s2 cob nad6 nad1 l2 rrnl16s v r rrns12s m c y w q l1 i a ,f g ,) | -h~-nad4,-v~-w,-w~a,i~-f,-g~e |
|  |  | T(t p nad4 nad4l s2 cob nad6 nad1 l2 rrnl16s v rrns12s m c y q l1 ,w ,) | |
| *F. acutargatus* | *Falcidens halanychi* |  |  |
| *F. acutargatus* | Caudofoveata ancestor | TDRL(t p f nad5 h nad4 nad4l s2 cob nad6 nad1 l2 rrnl16s v rrns12s m c y q g l1 ,r w e k cox3 i a ,) | atp6~-nad5,-v~-w,-w~a,i~-f,-f~-g,-g~e,k~s1 |
|  |  | TDRL(f w e k cox3 ,t p nad5 h nad4 nad4l s2 cob nad6 nad1 l2 rrnl16s v r rrns12s m c y q g l1 i a ,) | |
| *F. acutargatus* | Solenogastres ancestor | T(atp8 ,d ,) | cox2~d,d~atp8,atp8~atp6,atp6~-nad5,-nad4l~t,t~-s2,-nad6~p,p~-nad1,-l2~-l1,-l1~-rrns12s,-c~-q,-q~-y,-y~-rrnl16s,-v~-w,-w~a,a~r,r~i,i~-f,-f~-g,-g~e,e~cox3,cox3~k,k~s1,s1~n,nad3~nad2 |
|  |  | T(rrnl16s v ,rrns12s m c y q ,) |  |
|  |  | T(rrnl16s v rrns12s m c y w ,q ,) |  |
|  |  | T(y w ,q ,) |  |
|  |  | T(n nad3 ,s1 ,) |  |
|  |  | TDRL(t p f l1 e cox3 i ,nad5 h nad4 nad4l s2 cob nad6 nad1 l2 rrnl16s v r rrns12s m c y w q g a ,) | |
|  |  | TDRL(f nad5 h nad4 nad4l s2 cob nad6 nad1 l2 rrnl16s v r rrns12s m c y w q g l1 e k ,cox3 i a ,) | |
|  |  | TDRL(f nad5 h nad4 nad4l s2 cob nad6 nad1 l2 rrnl16s v rrns12s m c y w q g cox3 i a ,r l1 e ,) | |
|  |  | TDRL(rrnl16s v q ,rrns12s m c y ,) |  |
|  |  | I(e ) |  |
| *Ch. nitidulum* | *F. halanychi* | T(t p nad4 nad4l s2 cob nad6 nad1 l2 rrnl16s v r rrns12s m c y w q l1 i a ,f g ,) | -h~-f,-g~-w,-w~-nad4,-v~a,i~e |
|  |  | T(t p nad4 nad4l s2 cob nad6 nad1 l2 rrnl16s v rrns12s m c y q l1 ,w ,) | |
| *Ch. nitidulum* | Caudofoveata ancestor | T(t p nad4 nad4l s2 cob nad6 nad1 l2 rrnl16s v rrns12s m c y q l1 ,w g ,) | atp6~-nad5,-h~-f,-f~-g,-w~-nad4,-v~a,i~e,k~s1 |
|  |  | T(f ,nad5 h ,) |  |
|  |  | T(r i a ,e k cox3 ,) |  |
| *Ch. nitidulum* | Solenogastres ancestor | T(atp8 ,d ,) | cox2~d,d~atp8,atp8~atp6,atp6~-nad5,-h~-f,-f~-g,-g~-w,-w~-nad4,-nad4l~t,t~-s2,-nad6~p,p~-nad1,-l2~-l1,-l1~-rrns12s,-c~-q,-q~-y,-y~-rrnl16s,-v~a,a~r,r~i,i~e,e~cox3,cox3~k,k~s1,s1~n,nad3~nad2 |
|  |  | T(f ,nad5 h ,) |  |
|  |  | T(r e ,k cox3 i ,) |  |
|  |  | T(n nad3 ,s1 ,) |  |
|  |  | TDRL(t p f nad5 h nad4 nad4l s2 cob nad6 nad1 l2 rrnl16s v rrns12s m c y w q ,g l1 ,) | |
|  |  | TDRL(t p rrns12s m c y g l1 ,f nad5 h nad4 nad4l s2 cob nad6 nad1 l2 w q ,) | |
|  |  | TDRL(f nad5 h nad4 nad4l s2 cob nad6 nad1 l2 rrnl16s v ,rrns12s m c y w q ,) | |
|  |  | TDRL(r e cox3 ,k i ,) |  |
|  |  | I(r i ) |  |
|  |  | I(k cox3 ) |  |
|  |  | iT(r e k cox3 i ,a ,) |  |
| *F. halanychi* | *Scutopus robustus* | T(s2 ,cob ,) | atp6~-nad5,t~-s2,-s2~-cob,-cob~-nad6,-v~-w,-w~a,i~-f,-f~-g,-g~e,k~s1 |
|  |  | TDRL(t p f nad5 h nad4 nad4l s2 cob nad6 nad1 l2 rrnl16s v rrns12s m c y q g l1 ,r w e k cox3 i a ,) | |
|  |  | TDRL(f w e k cox3 ,t p nad5 h nad4 nad4l s2 cob nad6 nad1 l2 rrnl16s v r rrns12s m c y q g l1 i a ,) | |
| *F. halanychi* | *S. ventrolineatus* | TDRL(t p f nad5 h nad4 nad4l s2 cob nad6 nad1 l2 rrnl16s v rrns12s m c y q g l1 ,r w e k cox3 i a ,) | atp6~-nad5,-v~-w,-w~a,i~-f,-f~-g,-g~e,k~s1 |
|  |  | TDRL(f w e k cox3 ,t p nad5 h nad4 nad4l s2 cob nad6 nad1 l2 rrnl16s v r rrns12s m c y q g l1 i a ,) | |
| *F. halanychi* | Caudofoveata ancestor | TDRL(t p f nad5 h nad4 nad4l s2 cob nad6 nad1 l2 rrnl16s v rrns12s m c y q g l1 ,r w e k cox3 i a ,) | atp6~-nad5,-v~-w,-w~a,i~-f,-f~-g,-g~e,k~s1 |
|  |  | TDRL(f w e k cox3 ,t p nad5 h nad4 nad4l s2 cob nad6 nad1 l2 rrnl16s v r rrns12s m c y q g l1 i a ,) | |
| *F. halanychi* | Solenogastres ancestor | T(atp8 ,d ,) | cox2~d,d~atp8,atp8~atp6,atp6~-nad5,-nad4l~t,t~-s2,-nad6~p,p~-nad1,-l2~-l1,-l1~-rrns12s,-c~-q,-q~-y,-y~-rrnl16s,-v~-w,-w~a,a~r,r~i,i~-f,-f~-g,-g~e,e~cox3,cox3~k,k~s1,s1~n,nad3~nad2 |
|  |  | T(rrnl16s v ,rrns12s m c y q ,) |  |
|  |  | T(rrnl16s v rrns12s m c y w ,q ,) |  |
|  |  | T(y w ,q ,) |  |
|  |  | T(n nad3 ,s1 ,) |  |
|  |  | TDRL(t p f l1 e cox3 i ,nad5 h nad4 nad4l s2 cob nad6 nad1 l2 rrnl16s v r rrns12s m c y w q g a ,) | |
|  |  | TDRL(f nad5 h nad4 nad4l s2 cob nad6 nad1 l2 rrnl16s v r rrns12s m c y w q g l1 e k ,cox3 i a ,) | |
|  |  | TDRL(f nad5 h nad4 nad4l s2 cob nad6 nad1 l2 rrnl16s v rrns12s m c y w q g cox3 i a ,r l1 e ,) | |
|  |  | TDRL(rrnl16s v q ,rrns12s m c y ,) |  |
|  |  | I(e ) |  |
| Caudofoveata ancestor | Pruvotininae sp. | T(atp8 ,d ,) | cox2~d,d~atp8,atp8~atp6,atp6~-f,-nad4l~t,t~-s2,-nad6~p,p~-nad1,-l2~-l1,-l1~-rrns12s,-c~-q,-q~-y,-y~-rrnl16s,-v~-g,-g~-w,-w~e,e~cox3,cox3~k,k~a,a~r,r~i,i~s1,s1~n,nad3~nad2 |
|  |  | T(r rrns12s m c y w q g l1 e k cox3 i ,a ,) |  |
|  |  | T(k ,cox3 ,) |  |
|  |  | T(n nad3 ,s1 ,) |  |
|  |  | TDRL(t p ,f nad5 h nad4 nad4l s2 cob nad6 ,) |  |
|  |  | TDRL(rrnl16s v r rrns12s m c y w a ,q g l1 ,) |  |
|  |  | TDRL(rrnl16s v r q g a ,rrns12s m c y w l1 e k cox3 ,) |  |
|  |  | I(e ) |  |
| Caudofoveata ancestor | *Dorymenia* sp | T(atp8 ,d ,) | cox2~d,d~atp8,atp8~atp6,atp6~-f,-nad4l~t,t~-s2,-nad6~p,p~-nad1,-l2~-l1,-l1~-rrns12s,-c~-q,-q~-y,-y~-rrnl16s,-v~-g,-g~-w,-w~e,e~cox3,cox3~k,k~a,a~r,r~i,i~s1,s1~n,nad3~nad2 |
|  |  | T(rrnl16s v ,rrns12s m c y w q ,) |  |
|  |  | T(r k i ,cox3 a ,) |  |
|  |  | T(r i ,k cox3 a ,) |  |
|  |  | T(r a ,k ,) |  |
|  |  | T(k ,cox3 i ,) |  |
|  |  | T(n nad3 ,s1 ,) |  |
|  |  | TDRL(t p ,f nad5 h nad4 nad4l s2 cob nad6 ,) |  |
|  |  | TDRL(rrnl16s v q g ,y w ,) |  |
|  |  | I(rrnl16s v rrns12s m c y w q g ) |  |
|  |  | I(e ) |  |
|  |  | iT(rrnl16s v rrns12s m c y w q g ,l1 ,) |  |
| Caudofoveata ancestor | *W. argentea* | T(atp8 ,d ,) | cox2~d,d~atp8,atp8~atp6,atp6~-f,-nad4l~t,t~-s2,-nad6~p,p~-nad1,-l2~-l1,-l1~-rrns12s,-c~-q,-q~-y,-y~-rrnl16s,-v~-g,-g~-w,-w~e,e~cox3,cox3~k,k~a,a~r,r~i,i~s1,s1~n,n~nad3 |
|  |  | T(rrnl16s v rrns12s m c y w q g e ,l1 ,) |  |
|  |  | TDRL(t p ,f nad5 h nad4 nad4l s2 cob nad6 ,) |  |
|  |  | TDRL(rrnl16s v rrns12s m c y w ,q g ,) |  |
|  |  | TDRL(rrnl16s v q g ,rrns12s m c y ,) |  |
|  |  | TDRL(r s1 ,k i a ,) |  |
|  |  | TDRL(a n s1 ,r k i ,) |  |
|  |  | I(e ) |  |
|  |  | iT(r k i a n s1 ,cox3 ,) |  |
| Caudofoveata ancestor | *Falcidens acutargatus* | TDRL(t p nad5 h nad4 nad4l s2 cob nad6 nad1 l2 rrnl16s v r rrns12s m c y w q l1 i a ,f g e k cox3 ,) | atp6~-f,-f~-nad5,-v~-g,-g~-w,-w~e,k~a,i~s1 |
| Caudofoveata ancestor | *Falcidens halanychi* | TDRL(t p nad5 h nad4 nad4l s2 cob nad6 nad1 l2 rrnl16s v r rrns12s m c y w q l1 i a ,f g e k cox3 ,) | atp6~-f,-f~-nad5,-v~-g,-g~-w,-w~e,k~a,i~s1 |
| Caudofoveata ancestor | Solenogastres ancestor | T(atp8 ,d ,) | cox2~d,d~atp8,atp8~atp6,atp6~-f,-nad4l~t,t~-s2,-nad6~p,p~-nad1,-l2~-l1,-l1~-rrns12s,-c~-q,-q~-y,-y~-rrnl16s,-v~-g,-g~-w,-w~e,e~cox3,cox3~k,k~a,a~r,r~i,i~s1,s1~n,nad3~nad2 |
|  |  | T(rrnl16s v ,rrns12s m c y w q ,) |  |
|  |  | T(r k i ,cox3 a ,) |  |
|  |  | T(r i ,k cox3 a ,) |  |
|  |  | T(r a ,k ,) |  |
|  |  | T(k ,cox3 i ,) |  |
|  |  | T(n nad3 ,s1 ,) |  |
|  |  | TDRL(t p ,f nad5 h nad4 nad4l s2 cob nad6 ,) |  |
|  |  | TDRL(rrnl16s v q g ,y w ,) |  |
|  |  | I(rrnl16s v rrns12s m c y w q g ) |  |
|  |  | I(e ) |  |
|  |  | iT(rrnl16s v rrns12s m c y w q g ,l1 ,) |  |
| Caudofoveata ancestor | Polyplacophora ancestor | T(i ,n s1 ,) | -l1~-rrns12s,-c~-q,-q~-y,-y~-rrnl16s,-v~-g,-g~-w,-w~e,e~cox3,r~i,i~s1,s1~n,n~nad3,nad3~nad2 |
|  |  | T(i n nad3 ,s1 ,) |  |
|  |  | TDRL(rrnl16s v rrns12s m c y w ,q g ,) |  |
|  |  | TDRL(rrnl16s v q g ,rrns12s m c y ,) |  |
|  |  | I(e ) |  |
| Solenogastres ancestor | *S. robustus* | T(atp8 ,d ,) | cox2~atp8,atp8~d,d~atp6,atp6~t,t~p,p~-f,-nad4l~-s2,-s2~-cob,-cob~-nad6,-nad6~-nad1,-l2~-rrnl16s,-v~-rrns12s,-c~-y,-y~-w,-w~-q,-q~-g,-g~-l1,-l1~-e,-e~r,r~k,k~i,i~cox3,cox3~a,a~n,nad3~s1,s1~nad2 |
|  |  | T(t ,p f nad5 h nad4 nad4l ,) |  |
|  |  | T(t f nad5 h nad4 nad4l s2 cob nad6 ,p ,) |  |
|  |  | T(s2 ,cob ,) |  |
|  |  | T(r k i ,cox3 a ,) |  |
|  |  | T(r i ,k cox3 a ,) |  |
|  |  | T(r a ,k ,) |  |
|  |  | T(y w ,q g ,) |  |
|  |  | T(k ,cox3 i ,) |  |
|  |  | T(n nad3 ,s1 ,) |  |
|  |  | TDRL(rrnl16s v g ,rrns12s m c y q ,) |  |
|  |  | I(rrnl16s v rrns12s m c y w q g ) |  |
|  |  | I(e ) |  |
|  |  | iT(rrnl16s v rrns12s m c y w q g ,l1 ,) |  |
| Solenogastres ancestor | *S. ventrolineatus* | T(atp8 ,d ,) | cox2~atp8,atp8~d,d~atp6,atp6~t,t~p,p~-f,-nad4l~-s2,-nad6~-nad1,-l2~-rrnl16s,-v~-rrns12s,-c~-y,-y~-w,-w~-q,-q~-g,-g~-l1,-l1~-e,-e~r,r~k,k~i,i~cox3,cox3~a,a~n,nad3~s1,s1~nad2 |
|  |  | T(t ,p f nad5 h nad4 nad4l ,) |  |
|  |  | T(t f nad5 h nad4 nad4l s2 cob nad6 ,p ,) |  |
|  |  | T(r k i ,cox3 a ,) |  |
|  |  | T(r i ,k cox3 a ,) |  |
|  |  | T(r a ,k ,) |  |
|  |  | T(y w ,q g ,) |  |
|  |  | T(k ,cox3 i ,) |  |
|  |  | T(n nad3 ,s1 ,) |  |
|  |  | TDRL(rrnl16s v g ,rrns12s m c y q ,) |  |
|  |  | I(rrnl16s v rrns12s m c y w q g ) |  |
|  |  | I(e ) |  |
|  |  | iT(rrnl16s v rrns12s m c y w q g ,l1 ,) |  |
| Solenogastres ancestor | *Ch. nitidulum* | T(atp8 ,d ,) | cox2~atp8,atp8~d,d~atp6,atp6~t,t~p,p~-f,-f~-nad5,-h~-nad4,-nad4l~-s2,-nad6~-nad1,-l2~-rrnl16s,-v~-rrns12s,-c~-y,-y~-w,-w~-q,-q~-g,-g~-l1,-l1~-e,-e~r,r~k,k~i,i~cox3,cox3~a,a~n,nad3~s1,s1~nad2 |
|  |  | T(f ,nad5 h ,) |  |
|  |  | T(r k i ,cox3 ,) |  |
|  |  | T(n nad3 ,s1 ,) |  |
|  |  | TDRL(t f nad5 h nad4 nad4l s2 cob nad6 w g ,p nad1 l2 rrns12s m c y q l1 ,) | |
|  |  | TDRL(p f nad5 h nad4 nad4l nad1 l2 y g l1 ,t s2 cob nad6 rrnl16s v rrns12s m c w q ,) | |
|  |  | TDRL(nad1 l2 rrns12s m c w q g l1 ,t nad4 nad4l s2 cob nad6 rrnl16s v y ,) | |
|  |  | TDRL(k cox3 ,r e i ,) |  |
|  |  | I(r k cox3 i ) |  |
|  |  | iT(r e k cox3 i ,a ,) |  |
| Solenogastres ancestor | Caudofoveata ancestor | T(atp8 ,d ,) | cox2~atp8,atp8~d,d~atp6,atp6~t,t~p,p~-f,-nad4l~-s2,-nad6~-nad1,-l2~-rrnl16s,-v~-rrns12s,-c~-y,-y~-w,-w~-q,-q~-g,-g~-l1,-l1~-e,-e~r,r~k,k~i,i~cox3,cox3~a,a~n,nad3~s1,s1~nad2 |
|  |  | T(t ,p f nad5 h nad4 nad4l ,) |  |
|  |  | T(t f nad5 h nad4 nad4l s2 cob nad6 ,p ,) |  |
|  |  | T(r k i ,cox3 a ,) |  |
|  |  | T(r i ,k cox3 a ,) |  |
|  |  | T(r a ,k ,) |  |
|  |  | T(y w ,q g ,) |  |
|  |  | T(k ,cox3 i ,) |  |
|  |  | T(n nad3 ,s1 ,) |  |
|  |  | TDRL(rrnl16s v g ,rrns12s m c y q ,) |  |
|  |  | I(rrnl16s v rrns12s m c y w q g ) |  |
|  |  | I(e ) |  |
|  |  | iT(rrnl16s v rrns12s m c y w q g ,l1 ,) |  |
| Solenogastres ancestor | Polyplacophora ancestor | T(atp8 ,d ,) | cox2~atp8,atp8~d,d~atp6,atp6~t,t~p,p~-f,-nad4l~-s2,-nad6~-nad1,-l2~-rrnl16s,-g~-l1,-l1~-e,-e~r,r~k,k~i,i~cox3,cox3~a,a~n,n~nad3 |
|  |  | T(t ,p f nad5 h nad4 nad4l ,) |  |
|  |  | T(t f nad5 h nad4 nad4l s2 cob nad6 ,p ,) |  |
|  |  | T(rrnl16s v rrns12s m c y w q g ,l1 ,) |  |
|  |  | TDRL(k cox3 a ,r i n ,) |  |
|  |  | TDRL(cox3 n ,k i a ,) |  |
| Polyplacophora ancestor | Caudofoveata ancestor | T(y w ,q g ,) | -l1~-rrnl16s,-v~-rrns12s,-c~-y,-y~-w,-w~-q,-q~-g,-g~-e,-e~cox3,r~n,n~i,i~nad3,nad3~s1,s1~nad2 |
|  |  | TDRL(rrns12ts m c y q ,rrnl16s v g ,) |  |
|  |  | TDRL(i s1 ,n nad3 ,) |  |
|  |  | I(e ) |  |
|  |  | TDRL(rrns12s m c y q ,rrnl16s v g ,) |  |
| Polyplacophora ancestor | Solenogastres ancestor | T(atp8 ,d ,) | cox2~d,d~atp8,atp8~atp6,atp6~-f,-nad4l~t,t~-s2,-nad6~p,p~-nad1,-l2~-l1,-l1~-rrnl16s,-g~-e,-e~cox3,cox3~k,k~a,a~r,r~n,n~i,i~nad3 |
|  |  | T(rrnl16s v rrns12s m c y w q g ,l1 ,) |  |
|  |  | T(r a n ,k ,) |  |
|  |  | TDRL(t p ,f nad5 h nad4 nad4l s2 cob nad6 ,) |  |
|  |  | TDRL(r k i ,cox3 a n ,) |  |

**Supplementary Material S8: Gene orders of Solenogastres and Caudofoveata used for the CREx analyses.** Genes encoded on the opposite strand are preceded by -. rrnS and rrnL are listed as rrns12s and rrnl16s.

> Pruvotininae sp

cox1 cox2 atp8 d atp6 t p -f -nad5 -h -nad4 -nad4l -s2 -cob -nad6 -nad1 -l2 -rrnl16s -v r -rrns12s -m -c -y -w -q -g -l1 -e k cox3 i a n nad3 s1 nad2

> Kruppomenia borealis

cox1 cox2 atp8 d atp6 t p -f -nad5 -h -nad4 -nad4l -s2 -cob -nad6 -nad1 -l2 -rrnl16s -v -rrns12s -m -c -y -w -q -g -l1 -e r k i cox3 n a nad3 s1 nad2

> Dorymenia sp

cox1 cox2 atp8 d atp6 t p -f -nad5 -h -nad4 -nad4l -s2 -cob -nad6 -nad1 -l2 -rrnl16s -v -rrns12s -m -c -y -w -q -g -l1 -e r k i cox3 a n nad3 s1 nad2

> Wirenia argentea

cox1 cox2 atp8 d atp6 t p -f -nad5 -h -nad4 -nad4l -s2 -cob -nad6 -nad1 -l2 -rrnl16s -v -rrns12s -m -c -y -w -q -g -e -l1 -i -k -r -n -a -s1 cox3 nad3 nad2

> Scutopus robustus

cox1 cox2 d atp8 atp6 -f -nad5 -h -nad4 -nad4l t -cob -s2 -nad6 p -nad1 -l2 -l1 -rrns12s -m -c -q -y -rrnl16s -v -g -w e cox3 k a r i s1 n nad3 nad2

> Scutopus ventrolineatus

cox1 cox2 d atp8 atp6 -f -nad5 -h -nad4 -nad4l t -s2 -cob -nad6 p -nad1 -l2 -l1 -rrns12s -m -c -q -y -rrnl16s -v -g -w e cox3 k a r i s1 n nad3 nad2

> Falcidens acutargatus

cox1 cox2 d atp8 atp6 -nad5 -h -nad4 -nad4l t -s2 -cob -nad6 p -nad1 -l2 -l1 -rrns12s -m -c -q -y -rrnl16s -v -w a r i -f -g e cox3 k s1 n nad3 nad2

> Chaetoderma nitidulum

cox1 cox2 d atp8 atp6 -nad5 -h -f -g -w -nad4 -nad4l t -s2 -cob -nad6 p -nad1 -l2 -l1 -rrns12s -m -c -q -y -rrnl16s -v a r i e cox3 k s1 n nad3 nad2

> Falcidens halanychi

cox1 cox2 d atp8 atp6 -nad5 -h -nad4 -nad4l t -s2 -cob -nad6 p -nad1 -l2 -l1 -rrns12s -m -c -q -y -rrnl16s -v -w a r i -f -g e cox3 k s1 n nad3 nad2

> Laevipilina antarctica

cox1 cox2 atp8 atp6 t -f -nad5 -h -q -nad4 -nad4l -s2 -cob -nad6 -p -nad1 -l2 -l1 -rrnl16s -v -rrns12s -m -n -k -y -d -w -e g cox3 i r a nad3 s1 c nad2

> Polyplacophora ancestor

cox1 cox2 d atp8 atp6 -f -nad5 -h -nad4 -nad4l t -s2 -cob -nad6 p -nad1 -l2 -l1 -rrnl16s -v -rrns12s -m -c -y -w -q -g -e cox3 k a r n i nad3 s1 nad2

> Caudofoveata ancestor

cox1 cox2 d atp8 atp6 -f -nad5 -h -nad4 -nad4l t -s2 -cob -nad6 p -nad1 -l2 -l1 -rrns12s -m -c -q -y -rrnl16s -v -g -w e cox3 k a r i s1 n nad3 nad2

> Solenogastres ancestor

cox1 cox2 atp8 d atp6 t p -f -nad5 -h -nad4 -nad4l -s2 -cob -nad6 -nad1 -l2 -rrnl16s -v -rrns12s -m -c -y -w -q -g -l1 -e r k i cox3 a n nad3 s1 nad2

> Gastropoda ancestor

cox1 cox2 d atp8 atp6 -f -nad5 -h -nad4 -nad4l t -s2 -cob -nad6 -p -nad1 -l2 -l1 -rrnl16s -v -rrns12s -m -y -c -w -q -g -e cox3 k a r n i nad3 s1 nad2

> Mollusca ancestor

cox1 cox2 d atp8 atp6 -f -nad5 -h -nad4 -nad4l t -s2 -cob -nad6 p -nad1 -l2 -l1 -rrnl16s -v -rrns12s -m -c -y -w -q -g -e cox3 k a r n i nad3 s1 nad2
